# Regional gain and global loss of 5-hydroxymethylcytosine coexist in genitourinary cancers and regulate different oncogenic pathways

**DOI:** 10.1186/s13148-022-01333-4

**Published:** 2022-09-20

**Authors:** Jie Qi, Yue Shi, Yezhen Tan, Qi Zhang, Jianye Zhang, Jilu Wang, Cong Huang, Weimin Ci

**Affiliations:** 1 CAS Key Laboratory of Genomic and Precision Medicine, Beijing Institute of Genomics, Chinese Academy of Sciences and China National Center for Bioinformation, Beijing, 100101 China; 2 Institute of Urology, Peking University, Beijing, 100034 China; 3 Department of Urology, Peking University First Hospital, Beijing, 100034 China; 4 University of Chinese Academy of Sciences, Beijing, 100049 China; 5 Beijing Key Laboratory of Urogenital Diseases (Male) Molecular Diagnosis and Treatment Center, National Urological Cancer Center, Beijing, 100034 China; 6 Institute for Stem Cell and Regeneration, Chinese Academy of Sciences, Beijing, 100101 China

**Keywords:** 5**-**Hydroxymethylcytosine, Genitourinary cancers, hMeDIP-seq, Cancer stem cell-like cells, Vitamin C

## Abstract

**Background:**

DNA 5-hydroxymethylcytosine (5hmC) is produced by dynamic 5mC oxidation process contributing to tissue specification, and loss of 5hmC has been reported in multiple cancers including genitourinary cancers. However, 5hmC is also cell-type specific, and its variability may exist between differentiated tumor cells and cancer stem cells. Thus, cancer-associated changes in 5hmC may be contributed by distinct sets of tumor cells within the tumor tissues.

**Results:**

Here, we applied a sensitive immunoprecipitation-based method (hMeDIP-seq) to analyze 5hmC changes during genitourinary carcinogenesis (including prostate, urothelial and kidney). We confirmed the tissue-specific distribution of 5hmC in genitourinary tissues and identified regional gain and global loss of 5hmC coexisting in genitourinary cancers. The genes with gain of 5hmC during tumorigenesis were functionally enriched in regulating stemness and hypoxia, whereas were associated with poor clinical prognosis irrespective of their differences in tumor type. We identified that gain of 5hmC occurred in soft fibrin gel-induced 3D tumor spheres with a tumor-repopulating phenotype in two prostate cancer cell lines, 22RV1 and PC3, compared with conventional two-dimensional (2D) rigid dishes. Then, we defined a malignant signature derived from the differentially hydroxymethylated regions affected genes of cancer stem-like cells, which could predict a worse clinical outcome and identified phenotypically malignant populations of cells from prostate cancer tumors. Notably, an oxidation-resistant vitamin C derivative, ascorbyl phosphate magnesium, restored 5hmC and killed the cancer stem cell-like cells leading to apoptosis in prostate cancer cell lines.

**Conclusions:**

Collectively, our study dissects the regional gain of 5hmC in maintaining cancer stem-like cells and related to poor prognosis, which provides proof of concept for an epigenetic differentiation therapy with vitamin C by 5hmC reprogramming.

**Graphic Abstract:**

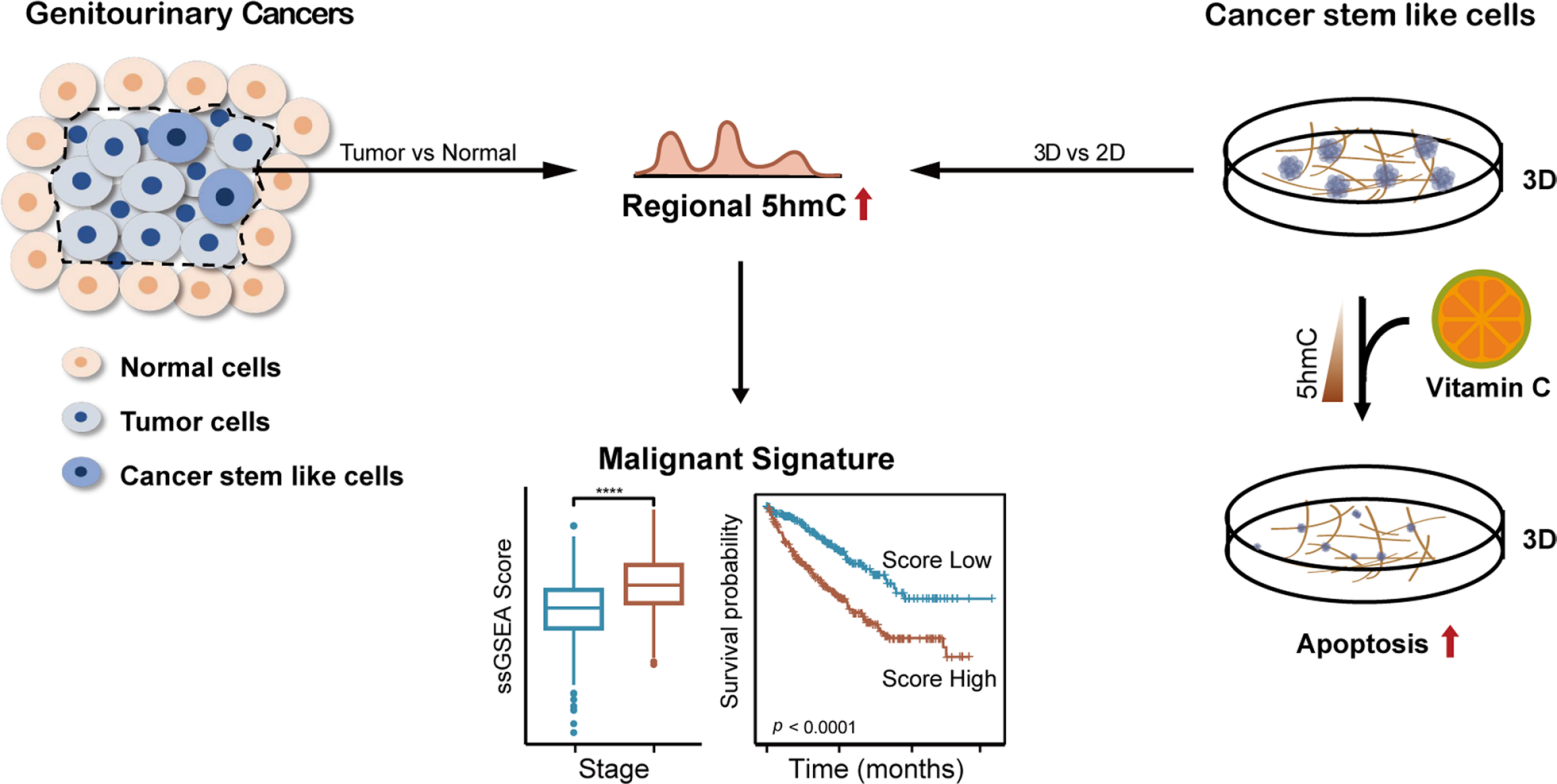

**Supplementary Information:**

The online version contains supplementary material available at 10.1186/s13148-022-01333-4.

## Background

Genitourinary (GU) cancers involving the kidney, bladder and prostate are characterized by high morbidity and mortality, leading to serious threats to human health [1]. Previous studies including ours have shown that epigenetic alterations, including 5**-**hydroxymethylcytosine (5hmC) in DNA, have functional consequences in GU tumorigenesis [2, 3]. Moreover, 5hmC is not a transient product of oxidation reactions via ten-eleven translocation (TET) enzymes depend on iron (II)/α-ketoglutarate to oxidize in the demethylation cycle but serves as a relatively stable DNA mark in the regulation of tissue-specific gene expression and is globally lost in many kinds of cancers [3–11]. Moreover, in kidney and bladder cancers, the 5hmC level is an independent prognostic marker, with lower levels of 5hmC associated with shorter overall survival [2, 3]. The conclusions emerging from these data suggest that loss of 5hmC is an epigenetic hallmark of cancer. Nonetheless, emerging evidence suggests that cancer is populated and maintained by tumor-repopulating cells with stem-like properties similar to those of adult tissue stem cells, which are a highly tumorigenic subpopulation of tumor cells [12–15]. Moreover, 5hmC affects the development and pluripotency of somatic stem cells [16–18]. Therefore, 5hmC variability may exist between differentiated tumor and cancer stem cells. Thus, cancer-associated changes in 5hmC may be contributed by distinct sets of tumor cells within the tumor tissues. We reasoned that specific enrichment of the 5hmC-modified DNA fragment could evaluate the 5hmC alterations both in small quantities of tumor-repopulating cells and in large quantities of differentiated tumor cells.

Thus, in this study, we applied a sensitive immunoprecipitation-based method (hydroxymethylcytosine DNA immunoprecipitation sequencing, hMeDIP-seq) to analyze 5hmC changes during genitourinary carcinogenesis. Strikingly, in addition to global 5hmC, we consistently identified regional gain of 5hmC in tumor tissues of GU cancers. The functional pathways of genes with 5hmC gain are enriched in stemness, hypoxia or cross talk with the immune response. We further confirmed that gain of 5hmC also occurred in cancer stem cell-like cells in soft 3D fibrin gel [19, 20] cultured prostate cancer cell lines. Notably, pretreatment of prostate cancer cells with the oxidation-resistant vitamin C derivative APM (ascorbyl phosphate magnesium) can kill cancer stem-like cells via raised the specific modified of 5hmC. Collectively, our findings provide new insight into 5hmC alterations in which both regional gain and global loss of 5hmC coexist in genitourinary cancers with different oncogenic pathways and provide a potential intervention strategy for the treatment of cancer stem-like cells with vitamin C and its derivatives.

## Methods

### Tissue collection

Ten pairs of normal and tumor tissues from renal cancer, prostate cancer and urothelial carcinoma patients were collected from Peking University First Hospital, and written informed consent was obtained from all patients. This study was approved by the Ethical Committee of Peking University First Hospital.

### Cell culture

The human prostate cancer cell lines PC3 and 22RV1 were purchased from the American Type Culture Collection (ATCC) and maintained in RPMI-1640 medium (Life Technologies, USA) supplemented with 10% fetal bovine serum (Life Technologies, USA), penicillin G (100 U/ml) and streptomycin (100 µg/ml) (Sigma-Aldrich, Germany). The human prostate and kidney normal cell lines RWPE-1 and HK-2 were purchased from ATCC and cultured in KM medium (Invitrogen, USA). The normal human urinary epithelial cell line SV-HUC-1 was obtained from ATCC and cultured in F-12 K medium (GIBCO, USA). All cells were maintained at 37 °C in humidified air containing 5% CO_2_.

### Cell proliferation assay, apoptosis assay and 3D soft fibrin gel cell culture of tumor cells

After 90% or 20% confluent cells were attached to the bottom of the culture dish, a quantitative amount of APM (Sigma-Aldrich, #A8960) was added to the culture medium. Cell proliferation was monitored and analyzed by IncuCyte (Essen Bioscience, USA). Apoptosis assay was performed by FACS with Annexin V staining.

The 3D culture was conducted according to a previously described method [20]. Cells were seeded mixed with 2 mg/mL fibrinogen (Searun Holdings Company, #SEA-133) and thrombin (0.1 U/mL, Searun Holdings Company, #SEA-135) in 24-well plates at a density of 2000 cells/well and cultured with different concentrations of APM. The cell culture plate was then moved into a 37 °C cell culture incubator for 30 min. Finally, 1640 medium containing 10% FBS and antibiotics was added.

### Animal experiment

All procedures involving mice and experimental protocols were approved by the University Institutional Animal Care and Use Committee (IACUC). The study is compliant with all relevant ethical regulations regarding animal research. Four- to six-week-old male nu/nu mice were used in the mouse experiment. Mice were randomized into two groups. Prostate cancer cell spheroids were selected from 3D 90-Pa soft fibrin gels. For conventional 2D cell culture, tumor cells were maintained in rigid dishes with complete culture medium. Then, the cells were pipetted into single cells, and the cell number was counted under a microscope. The cells were then suspended in PBS at 30 thousand per 100 µl. Thirty thousand tumor-repopulating cells (TRCs) were intravenously injected subcutaneously into each nu/nu mouse. Ten days later, the columns of inoculated mice were measured every three days. The mice were euthanized and examined for prostate tumor formation at Day 40.

### DNA/RNA extraction

Genomic DNA was extracted from fresh patient tissues and cell lines using QIAamp DNA Mini Kits (QIAGEN, Germany). FFPE samples were extracted using QIAamp DNA FFPE Tissue Kits (QIAGEN, Germany) according to the manufacturer’s instructions. RNA was extracted from the prostate cancer cells using TRIzol. The DNA/RNA concentration was measured using a Qubit fluorometer (Life Technology, USA).

### RT-qPCR and hMeDIP-qPCR

Total RNA was extracted from cells using TRIzol reagent (Invitrogen), and the quantity and quality of RNA were determined by using a NanoDrop 1000. Single-strand cDNA was synthesized using the RevertAid First Strand cDNA Synthesis Kit (Thermo Scientific, #K16225). Real-time PCR was performed using the SYBR FAST PCR master mix (Vazyme, #Q712-02) on a Multiplex Quantitative PCR System (Bio-Rad CFX96 TOUCH/GelDoc XR +). The ΔCt method was used to analyze mRNA levels relative to GAPDH. 1 µg genomic DNA was sonicated to 300–400 bp using Covaris, heat denatured and set aside 10% as the input. The remainder was divided equally, and add 0.5 µg 5hmC antibody, overnight at 4 °C. The complexes were captured by the protein G beads and were subsequently used for hMeDIP**-**qPCR analysis. The primer sequences are listed in Additional file 9: Table S1.

### Dot blot

The dot blot procedure was performed as previously described [21], with minor modifications. Briefly, the genomic DNA samples were denatured in 0.4 M NaOH, 10 mM EDTA at 95 °C for 10 min. Then, the samples were neutralized by adding an equal volume of cold 2 M ammonium acetate (pH 7.0) on ice. Denatured DNA samples were spotted on a nitrocellulose membrane in a Bio-Dot apparatus (Bio–Rad) with twofold serial dilutions according to the manufacturer’s instructions. The membrane was washed with 2X SSC buffer, air-dried and vacuum-baked at 80 °C for 2 h, blocked with 5% milk in PBST (PBS buffer with 0.1% Tween-20) buffer at room temperature for 1 h, incubated with the anti-5hmC antibody (Active Motif, #39,769, 1:10,000) overnight at 4 °C and then washed three times with PBST buffer. After incubation with a horseradish peroxidase-conjugated goat anti-rabbit secondary antibody (1:5,000) at room temperature for 1 h, the membrane was washed three times with PBST and visualized by chemiluminescence. Staining of the membranes by methylene blue was used to indicate equal loading.

### HMeDIP-seq and RNA-seq Library preparation

The hMeDIP-seq procedure has been described in a recent study [3]. The sequencing libraries were prepared with 2 µg of genomic DNA ligated to PE adaptors (Illumina, USA) followed by 5hmC antibody capture for immunoprecipitation. The captured fragments were amplified and quantified on an Agilent 2100 Bioanalyzer before cluster generation and sequencing on a HiSeq 3000 according to the manufacturer’s protocols.

The RNA sequencing libraries were prepared with 40 µg of total RNA ligated to PE adaptors (Illumina, USA) followed by enrichment by magnetic beads with oligo (dT). The captured fragments were amplified and quantified on an Agilent 2100 Bioanalyzer before cluster generation and sequencing on a HiSeq 3000 according to the manufacturer’s protocols.

### Sequencing data processing

Reads from the 5hmC antibody grab were aligned to the *Homo sapiens* reference genome (hg19, UCSC) using BWA version 0.7.12 [22] with default settings. All duplicates, unmapped reads, reads with more than 10 mismatches and nonuniquely mapped reads were removed using SAMtools version 1.3.0 [22]. Snapshots of the data were constructed using the Integrative Genomics Viewer (IGV) [23]. MACS2 [24] was used for peak identification in individual replicates, and peaks with < twofold enrichment were discarded to reduce the noise during data analysis. The per-base genomic coverage was obtained using bedtools [25]. Then, MACS2 peaks were annotated by the CEAS program [26] in the hg19 human genome.

For RNA-seq, reads were aligned to the *Homo sapiens* reference genome (hg19, UCSC) using HISAT2 [27] with default settings. Reads with more than 20 mismatches and nonuniquely mapped reads were removed using SAMtools version 1.3.0 [22]. Total read counts for each protein-coding gene were extracted using HTSeq (version 0.6.0) [28]. R package DESeq2 [29] was applied to identify differentially expressed genes with fold change ≥ 1.5 and FDR < 0.05.

### Distribution and cluster analysis

A list of functional DNA elements was defined according to genomic locations: promoter (upstream 1000 bp region from the TSS of each gene), gene body (the region of each gene without a promoter or terminal region) and intergenic regions (the remaining genome region lacking any promoter, terminal region and gene body region). To estimate the enrichment of 5hmC signals in and around functional DNA elements, normalized coverage over 20-bp genomic bins was estimated based on aligned reads using bamCoverage of deeptools [30]. The normalized coverage was then summarized and visualized as heatmap using the function computeMatrix and plotProfile. Sample clustering was performed according to gene FPKM values of the hMeDIP-seq and RNA-seq data.

### Malignant signature scoring and survival analysis

Enrichment scores of hypo- and hyper-DhMR gene signatures were estimated using ssGSEA which was implemented by the GSVA R package [31]. In survival analysis, samples with incomplete clinical data were removed. All events were considered regardless of radiological or pharmaceutical treatment received. Kaplan–Meier survival analyses were performed using the survival and survminer R packages. Depending on the signature being investigated, patients were categorized into a “high” or “low” group of estimated enrichment scores using the median as cutoffs [32]

### Phenotype-guided single-cell subpopulation identification by Scissor

The Scissor R package was used to identify cell subpopulations associated with given phenotypes from single-cell RNA-seq data [33]. Here, we analyzed a single-cell RNA-seq dataset comprising 36,424 cells of PRAD from a published study [34]. Bulk gene expression profiles of 495 PRAD samples were collected from TCGA, and the phenotypes were determined by the malignancy score. Briefly, Scissor first uses quantile normalization on the single-cell and bulk expression data to remove the underlying batch effect. Subsequently, a Pearson correlation matrix was calculated for each pair of cells and bulk samples to quantify the similarity between the single-cell and bulk data. According to the correlation of single-cell and corresponding bulk data, we denoted the selected cells based on Scissor-positive (Scissor^+^) cells and Scissor-negative (Scissor^−^) cells, which were positively and negatively associated with the phenotype of interest, respectively. Finally, the scissor-selected cells were further investigated by several downstream analyses, such as differential expression gene and functional enrichment analyses, to reveal the underlying biological mechanisms of the selected cell subpopulations.

### Annotation

The peaks of hypo- or hyper-hydroxymethylated regions were annotated using Genomic Regions Enrichment of Annotations Tool (GREAT) [35] which assigns biological processes directly. Functional annotation analysis was conducted in the GO analysis via Metascape [36] software for annotation and plotted the most significant (− log10 (*P*-value)) of the overlapped gene lists to show their statistical significance.

### Statistical analysis

All analysis was performed using R version 4.0.3. Continuous variables were compared using the t test or Mann–Whitney *U* test. Statistical significance was defined as *P < 0.05, **P < 0.01 and ***P < 0.001.

## Results

### Genome-wide 5hmC profiling of the genitourinary system in health and cancer

**Fig. 1:**
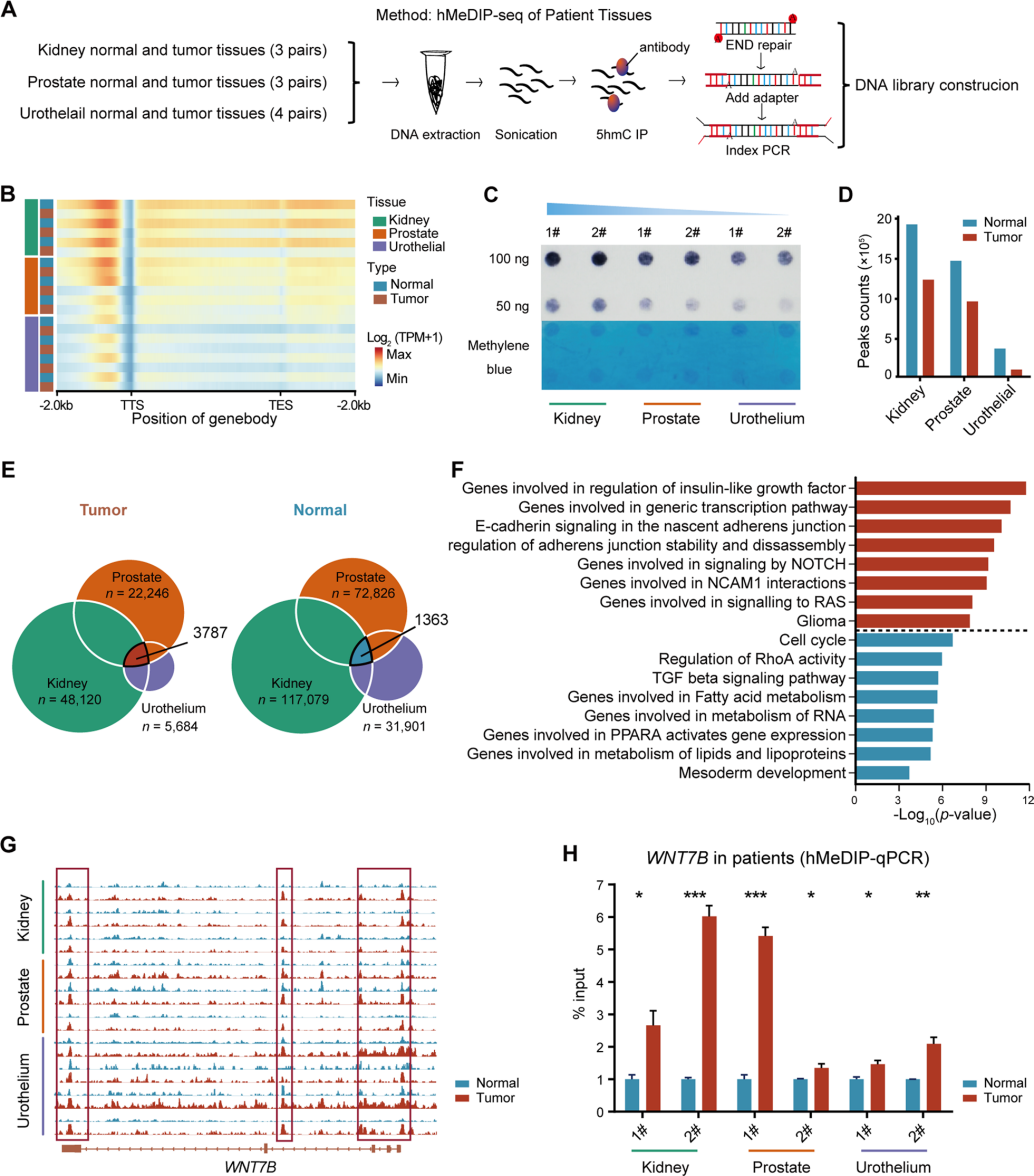
Genome-wide 5hmC profiling of the genitourinary system in heath and cancer. **A** Schematic diagram showing the genome-wide 5hmC sequencing of healthy and cancerous genitourinary tissues. **B** Meta-gene plot of 5hmC profiles in genitourinary tissues. The color range indicates log_2_ (TPM + 1) values. TSS, transcription start site. TES, transcription end site. **C** Dot blot showing distinct 5hmC levels in normal genitourinary tissues including the kidney, the prostate and the urothelium (top). Methylene blue (MB) staining was used as a DNA loading control (bottom). **D** Bar plot displaying numbers of total 5hmC peaks identified in normal genitourinary tissues and genitourinary tumors. **E** Venn diagrams showing overlapping normal-specific peaks in genitourinary tissues (left) and the overlap of tumor-specific 5hmC peaks (right). The intersections are peaks common to healthy genitourinary tissues (left) and genitourinary tumors (right). **F** Gene Ontology (GO) analysis of the common 5hmC peaks. **G** Genome browser tracks depicting the 5hmC level of *WNT7B* in healthy and cancerous genitourinary tissues including the kidney, the prostate and the urothelium. **H** Bar plot showing the 5hmC level of *WNT7B* measured by hMeDIP-qPCR in matched normal and tumor samples of the kidney, the prostate and the urothelium. Primers were designed at the positions indicated by red boxes in (**G**). Error bars represent mean ± standard deviation. *P* values were produced with *t* test

To precisely profile genome-wide 5hmC patterns in the genitourinary system, we performed hMeDIP-seq of 10 tumors (3 kidney cancer, 3 prostate cancer and 4 urothelial cancer) and paired normal tissues (Fig. 1A). As expected, unsupervised clustering uncovered the existence of tissue-specific 5hmC patterns in healthy and cancerous GU tissues (Additional file 1: Fig. S1A, B). Interestingly, we found that normal renal tissues had the highest 5hmC level, while normal urothelial tissues showed the lowest 5hmC modification at the gene body and 1-kb flanking region (Fig. 1B, Additional file 1: Fig. S1C). The global 5hmC pattern of healthy genitourinary samples was further validated by dot blot using normal tissues and three normal GU cell lines (Fig. 1C and Additional file 1: Fig. S1D). Consistent with previous reports [2, 3, 7], the meta-gene analysis suggested a global loss of 5hmC in all of the 3 types of GU cancers compared with the matched normal tissues, which was also confirmed by the reduced number of valid 5hmC peaks (Fig. 1B–D). By combining the normal and tumor peaks, we found that the kidney cancer had decreased hydroxymethylation at the 3’-UTR, TES and introns, while the urothelial cancer had a widespread dehydroxymethylation (Additional file 1: Fig. S1E).

Tumors are disease of high heterogeneity. Although cancers of the kidney, prostate and urothelium had distinct DNA 5hmC landscape, we still wondered whether there is a universal change of biological process underlying the tumorigenesis of different GU cancers. Therefore, we listed the 5hmC peaks for the three normal and cancerous GU tissues and excluded those consecutively active for each type of tissue (Additional file 2: Fig. S2A, B). The remaining normal and tumor-specific peaks were intersected (Additional file 2: Fig. S2A–D). To this end, we identified 3787 and 1363 5hmC peaks that were shared by cancerous and healthy GU tissues, respectively (Fig. 1E). While 5hmC peaks shared by normal GU tissues influenced genes involving tissue development and homeostasis, common 5hmC peaks in GU cancers were enriched for several oncogenic GO terms including the regulation of insulin-like growth factor and Notch signaling[37] (Fig. 1F). Specifically, a consecutive hydroxymethylation at the promoter and gene body of *HOXB8* and *HOXB9* was seen in normal GU tissues (Additional file 2: Fig. S2E, F). Similarly, the promoter of the oncogene *WNT7B* was uniformly hydroxymethylated in GU cancers but not normal tissues, which could also be validated by hMeDIP-qPCR (Fig. 1G, H). Collectively, these results offered a genome-wide 5hmC landscape of the genitourinary system in both health and cancer, which revealed common 5hmC alterations contributing to the progression of GU tumors.

### Aberrant gain and loss of 5hmC coexist in genitourinary cancers and are associated with different oncogenic pathways

**Fig. 2:**
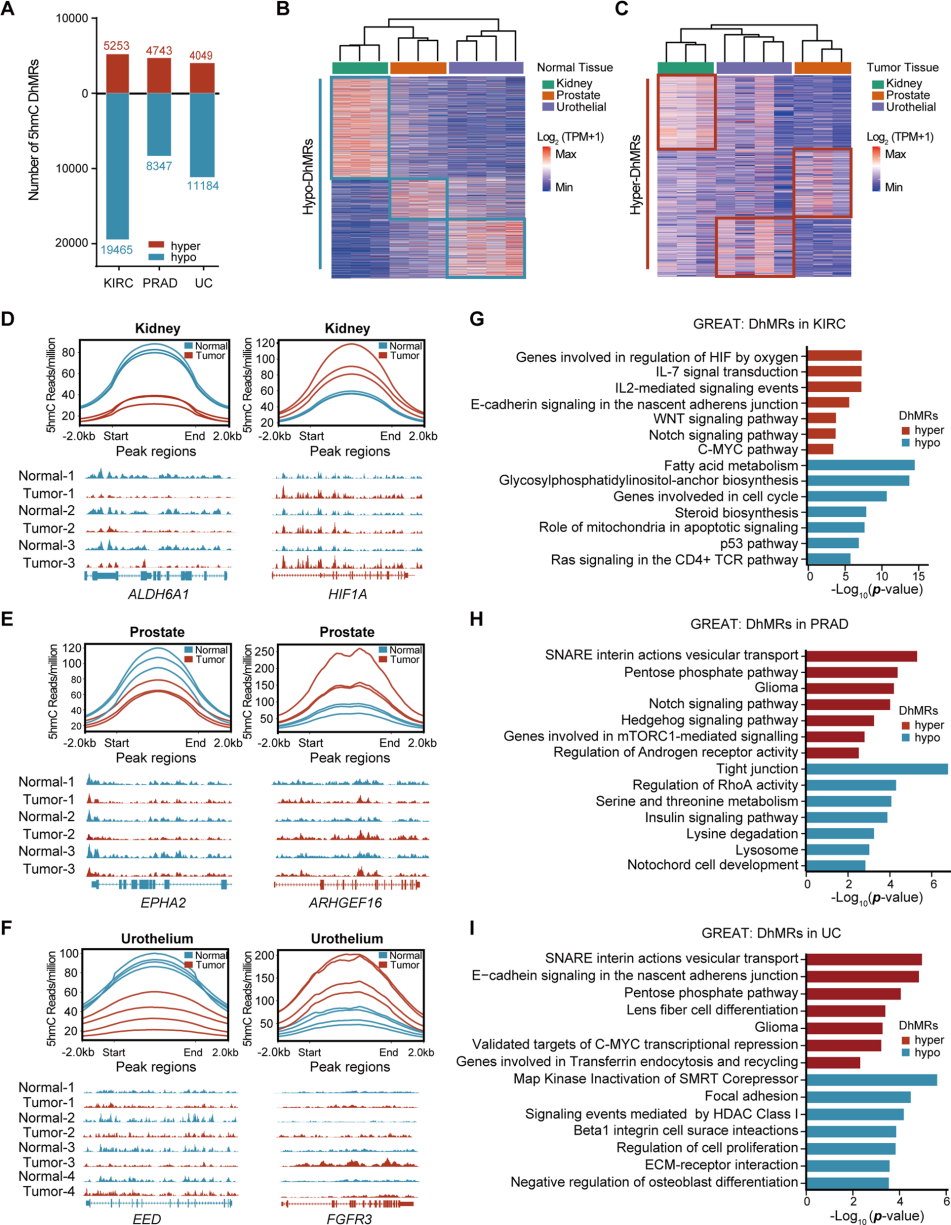
Aberrant gain and loss of 5hmC coexist in genitourinary cancers and are associated with different oncogenic pathways. **A** Bar plot displaying numbers of hyper- and hypo-DhMRs identified in kidney, prostate and urothelial tumors compared with healthy controls. **B, C** Heatmaps showing the clustering of normal genitourinary samples based on hypo-DhMRs (**B**) and the clustering of genitourinary tumors based on hyper-DhMRs (**C**). Colors indicate normalized read counts of hMeDIP-seq. **D**–**F** Genome browser tracks depicting 5hmC levels of hypo (left)- and hyper-DhMRs (right) in kidney cancer (**D**), prostate cancer (**E**) and urothelial cancer (**F**). **G**–**I** Functional significance of hypo- and hyper-DhMRs of kidney cancer (**G**), prostate cancer (**H**) and urothelial cancer (**I**)

To systemically investigate the disruption of DNA hydroxymethylation during tumorigenesis, we identified differentially hydroxymethylated regions (DhMRs) in cancerous genitourinary tissues (Additional file 3: Fig. S3A). A total of 24,718, 13,090, and 15,233 peaks were subsequently obtained as DhMRs for the kidney, prostate, and urothelial cancers, of which the majority were hypo-DhMRs (Fig. 2A, Additional file 3: Fig. S3B, and Additional file 10: Table S2). In addition, the preference of hypo-hydroxymethylation over hyper-hydroxymethylation was observed at almost all autosomes in the three GU cancers we investigated (Additional file 3: Fig. S3C). Notably, both hypo- and hyper-DhMRs we collected were highly tissue-specific (Fig. 2B, C).

The annotation of hyper-DhMRs suggested the presence of substantial differently enriched gene sets in the three GU cancers (Fig. 2G–I). In kidney cancer, the hyper-DhMRs were significantly associated with hypoxia (Fig. 2G). Consistently, hyper-hydroxymethylation of *HIF1A* was observed (Fig. 2D). In prostate cancer, there was a strong enrichment of androgen signaling encompassing hyper-hydroxymethylated *ARHGEF16* (Fig. 2E–H). Additionally, the urothelial carcinoma harbored elevated 5hmC at the promoter and gene body of *FGFR3*, which was reported to play a central role in its tumorigenesis [38] (Fig. 2F–I). However, hypo-DhMRs were linked to genes involving biosynthesis and metabolism irrespective of tumor type (Fig. 2G–I).

Functionally, we found that these DhMRs affected genes were positively changed in mRNA expression during the tumorigenesis of the corresponding GU cancers by analyzing the RNA-seq data from The Cancer Genome Atlas (TCGA) (Additional file 4: Fig. S4A–C). Collectively, our results suggested that the tumorigenesis in the genitourinary system is accompanied by both gain and loss of DNA 5hmC, which is tissue-specific but also indicative of a universal tumor biology.

### Genes associated with 5hmC alterations predict clinical outcome in genitourinary cancers

**Fig. 3:**
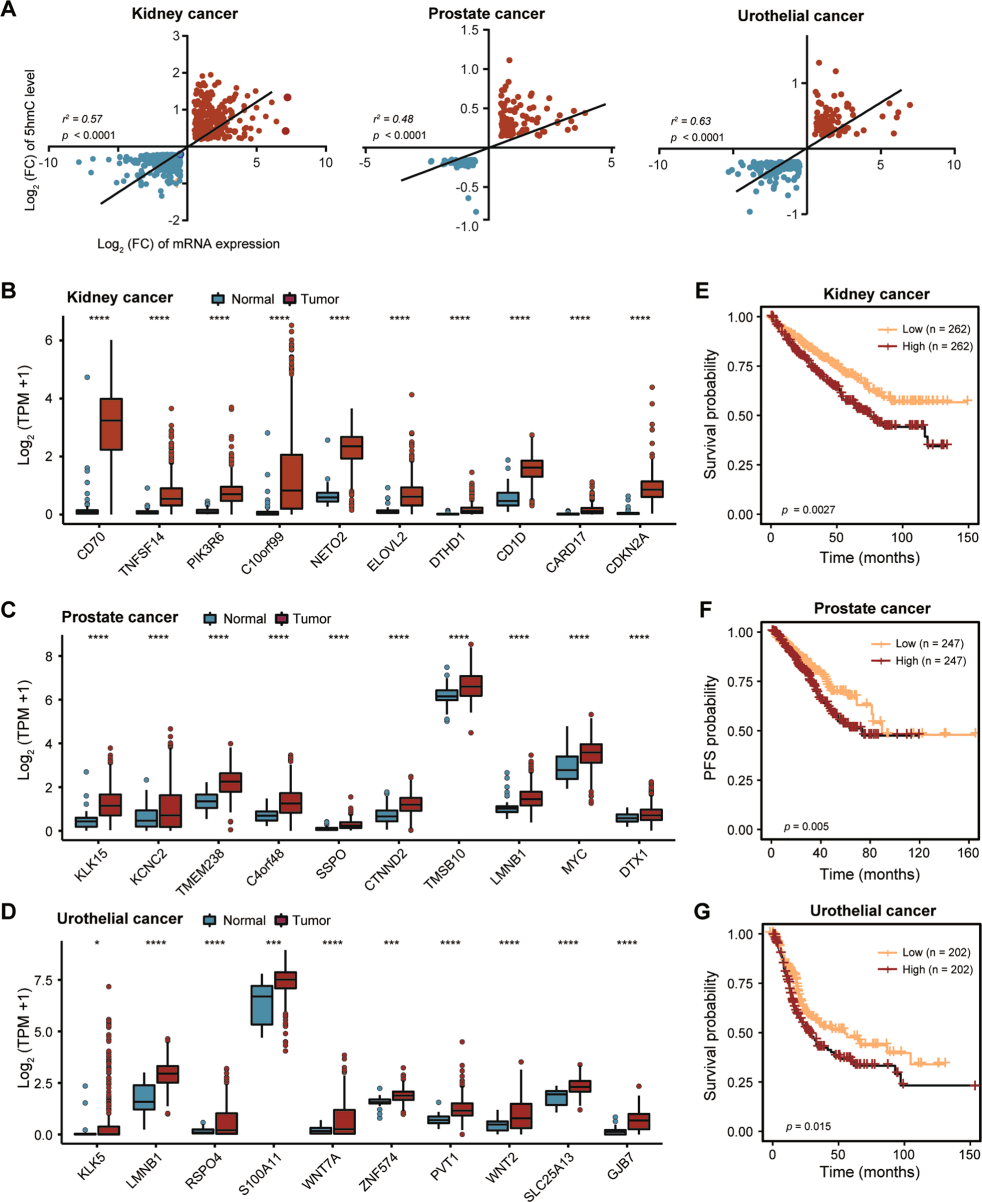
Genes associated with 5hmC alterations predict clinical outcome in genitourinary cancers. **A** Scatter plots showing the positive correlation between 5hmC alterations and mRNA expression changes in genitourinary cancers. **B–D** Box plots displaying the expression of hyper-DhMR associated genes with the most transcriptional changes compared to normal tissues in the TCGA KIRC (**B**), PRAD (**C**) and BLCA (**D**) cohorts. *P* values were determined by the Student’s *t* test. ****P < 0.0001; ***P < 0.001; **P < 0.01;*P < 0.05. **E–G** Kaplan–Meier plots of the relationship between patient overall survival, progression-free survival and hyper-hydroxymethylated signature scores within the TCGA KIRC (**E**), PRAD (**F**) and BLCA (**G**) cohorts. Patients were stratified by the score median. *P* values were produced using log rank test

State of 5hmC is closely related to gene expression. By integrating the RNA-seq data of GU cancers from TCGA with our hMeDIP-seq data, we found a significant positive correlation between 5hmC alterations and changes in mRNA expression during the tumorigenesis of renal cancer, prostate cancer and urothelial carcinomas (Fig. 3A and Additional file 5: Fig. S5A, Additional file 11: Table S3). Since many gene signatures have been reported to be prognostic biomarkers for GU cancers [39], we sought to evaluate the efficacy of genes associated with hypo- and hyper-hydroxymethylation in predicting patient survival. To generate such gene signatures, we collected these hypo- and hyper-DhMRs in each type of cancer and annotated them to genes, from which top 10 candidates were selected according to the expression fold change in tumors (Fig. 3B–D, Additional file 5: Fig. S5B–D). Surprisingly, patients with high hyper-hydroxymethylated signature had significantly worse overall survival or progression-free survival (PFS) in the TCGA KIRC, BLCA and PRAD cohorts, which illustrates that genes associated with 5hmC alterations are of potential clinical significance (Fig. 3E–G, Additional file 5: Fig. S5E–G).

### Increased stemness and DNA 5hmC in 3D-cultured prostate cancer cells

**Fig. 4:**
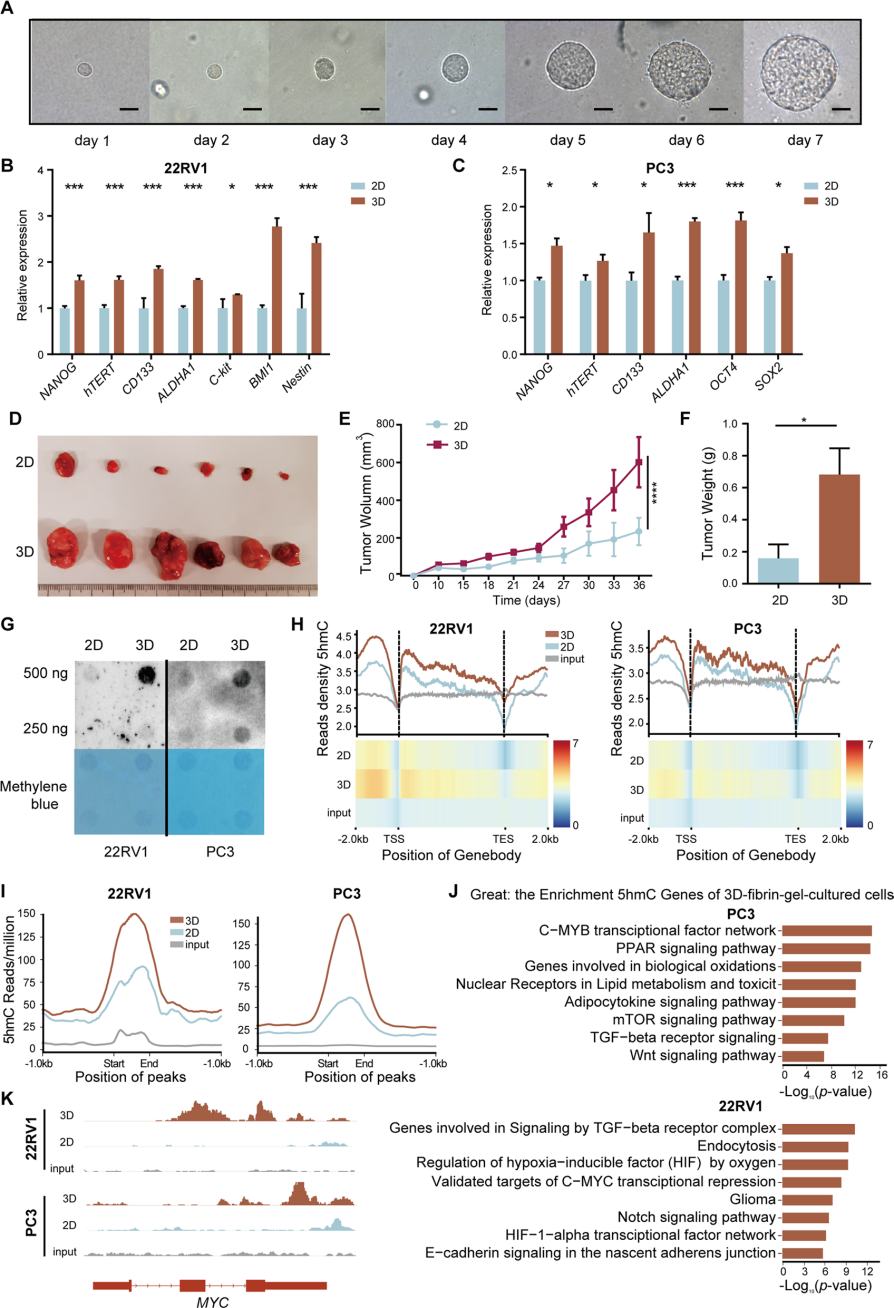
Increased stemness and DNA 5hmC in 3D-cultured prostate cancer cells. **A** Formation and growth of spheroids by 3D-cultivating 22RV1 cells for 7 consecutive days; scale bar = 10 µm. **B**, **C** Quantification of the stem cell marker expression in 22RV1 and PC3 cell lines by real-time PCR. Cells cultured in 2D rigid dishes were used as control. **D**–**F** Subcutaneous xenografts (**D**) derived from 2D- to 3D-cultured 22RV1 cells, the growth kinetics (**E**) determined by measuring tumor volume and corresponding tumor weights (**F**). Each group *n* = 6. **G, H** Dot blot (**G**) and hMeDIP-seq (**H**) showing the 5hmC alteration introduced by the 3D culture in prostate carcinoma cell lines 22RV1 and PC3. **I** 5hmC density at hyper-DhMRs shared by 3D-cultured 22RV1 (left) and PC3 (right) cells. **J** Functional relevance of the hyper-hydroxymethylation introduced by 3D-culturing of 22RV1 (left) and PC3 (right) cells. **K** Genome browser tracks depicting the 5hmC surge of *MYC* attributed to 3D-culturing. In (**B**), (**C**), (**E**) and (**F**), error bars represent mean ± standard deviation. *P* values were produced with *t* test. ****P < 0.0001; ***P < 0.001; **P < 0.01;*P < 0.05.

Cancer progression to an aggressive phenotype often co-opts gain of stem cell-like states and epigenome reprogramming [18, 40, 41]. To gain more insights into such epigenetic reprograming processes, we applied a previously described mechanical method to obtain aggressive cancer stem-like cells by culturing prostate cancer cells in fibrin matrices of ∼90 Pa stiffness [42, 43], which resulted in the formation and growth of multicellular tumor spheroids (Fig. 4A). Using real-time reverse transcription-PCR (RT-qPCR), we discovered that several canonical stem cell makers, including *NANOG*, *CD133*, and *OCT4*, were up-regulated in the soft-fibrin-gel-selected cancer cells (Fig. 4B, C), which confirmed their cancer stem cell identity. Additionally, a significant increase in tumor volume and mass for the 3D-cultured group compared with two-dimensional (2D) controls was observed in the mouse xenograft model, suggesting that 3D-cultured prostate cancer cells acquired an aggressive cancer stem cells (CSCs) phenotype (Fig. 4D–F).

Having generated CSCs from prostate cancer cells, we sought to better resolve cellular 5hmC dynamics introduced by 3D-culture. Interestingly, using dot blot we identified a global increase of 5hmC in tumor spheres induced by 3D-culturing (Fig. 4G), which was also evident in subsequent hMeDIP sequencing (Fig. 4H, I). A total of 1,214 and 1,671 genes were identified as being hyper-hydroxymethylated in the 3D-cultured 22RV1 and PC3 cells compared with 2D controls, respectively (Additional file 12: Table S4). By annotating them to relevant biological processes, we noticed significant enrichments in signaling pathways involving mTOR, WNT, Notch, C-myc and hypoxia, which might contribute to the maintenance of stemness and promote tumor progression (Fig. 4J-K). Intriguingly, these enriched GO terms coincide well with what describes hyper-DhMRs in prostate tumors (Fig. 2H), suggesting that specific 5hmC gains might facilitate the initiation and progression of prostate cancer and are associated with cancer cell stemness.

### Stemness-associated 5hmC gain defines a highly malignant cancer cell population in prostate tumors

We next sought to further investigate the clinical implication of the stemness-associated 5hmC gain. By coupling with RNA-seq, we found that the 3D-culturing gave rise to synchronous enhancements in the gene expression and DNA 5hmC of *KITLG*, *SOX21*, *CDH1* and *WNT9A*, which are known stem cell maintainers [44–46] (Additional file 6: Fig. S6A). Moreover, enrichment scores of gene sets comprising simultaneously up-regulated and hyper-hydroxymethylated genes in 3D-cultured PC3 (*n* = 166) and 22RV1 cells (*n* = 171) positively correlated with the Gleason score in the TCGA PRAD cohort (Additional file 6: Fig. S6A-B), which resembles to the adult stem cell signature, naive hESC signature and primed hESC signature described in previous studies [47], implying the critical role of the regional 5hmC gain in the tumorigenesis and progression of prostate cancer (Additional file 6: Fig. S6C).

**Fig. 5:**
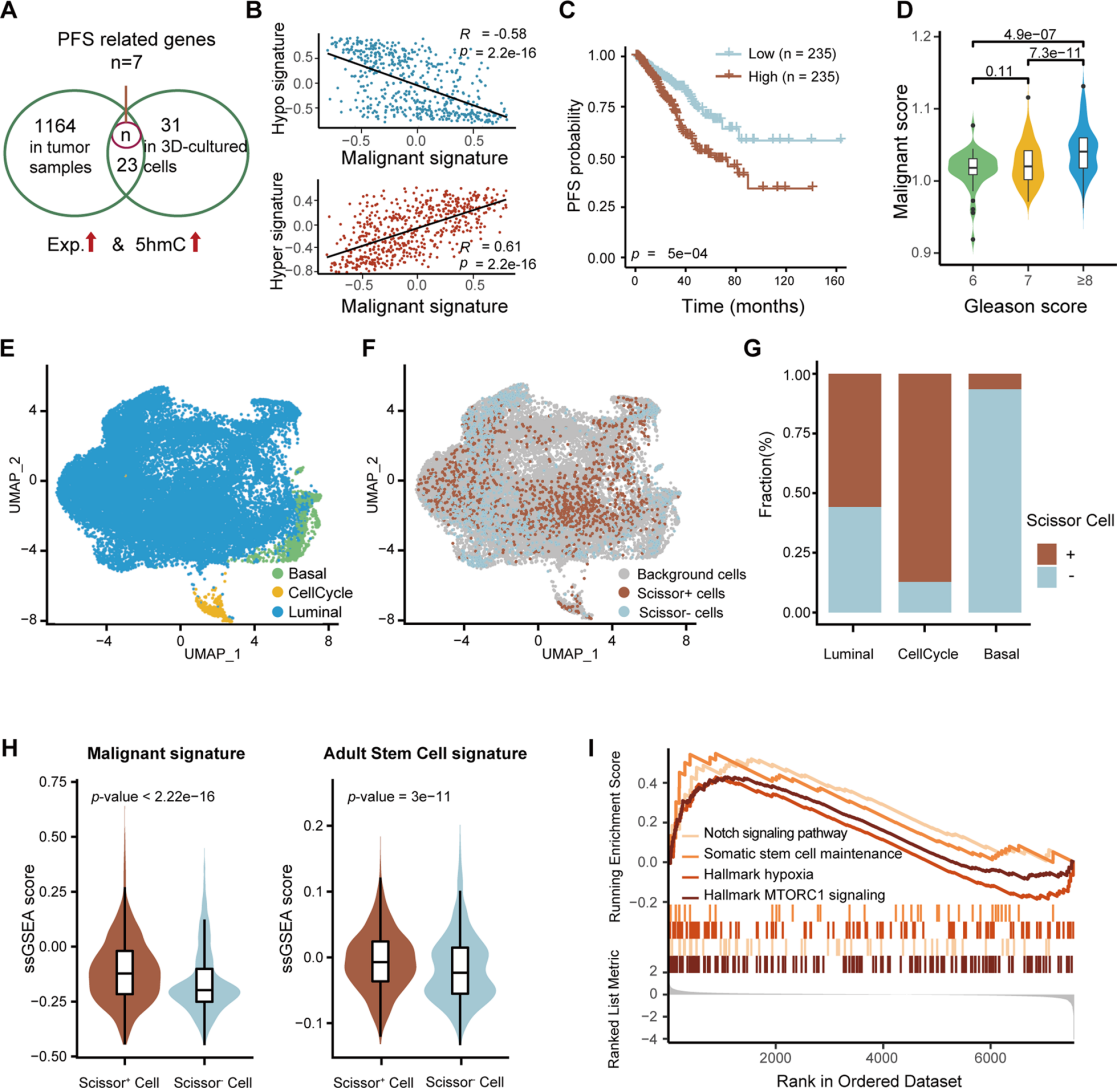
Quantification of the stemness-associated 5hmC gain with a malignant signature. **A** Venn diagram showing 30 genes obtaining increased 5hmC and expression in prostate tumors and 3D-cultured prostate tumor cells. Seven genes are highlighted for being PFS-related in the TCGA PRAD cohort. **B** Pearson’s correlation analysis showing the malignant signature is negatively associated with the hypo-hydroxymethylation signature and vice versa. **C** Kaplan–Meier plot of the relationship between patient PFS and the malignant signature score within the TCGA PRAD cohort. Patients were stratified by the score median. *P* values were produced using log rank test. **D** Violin plots showing estimated malignant signature scores of patients stratified by the Gleason score. **E** UMAP projections of 23,674 prostate cancer cells, color-coded by subtypes. **F** UMAP visualization of the Scissor^+^ cells which are predicted to possess a higher malignant signature. **G** Bar plot depicting the fraction of Scissor^+^ cells in the 3 subtypes of prostate tumor cells. **H** Violin plots showing differences in the malignant signature (left) and the adult stem cell signature (right) between the Scissor^+^ and Scissor^−^ populations. **I.** GSEA displaying enriched gene sets in Scissor^+^ cells. In (**D**) and (**H)** *P* values were produced with *t* test. In (**B**), (**C**), (**E**) and (**F)**, error bars represent mean ± standard deviation. *P* values were produced with Mann–Whitney *U* test.

Among genes exhibiting hyper-hydroxymethylation and elevated expression in both PRAD tissues and 3D-cultured cells, 7 genes (*NVL*, *LUC7L3*, *TTC13*, *RHOU*, *CABLES2*, *ZFPM1* and *ZSWIM4*) were associated with poor PFS in the TCGA PRAD cohort (Fig. 5A). Using hMeDIP-qPCR and RT-qPCR, we validated that higher 5hmC and expression levels of these genes were readily detected in 3D-cultured 22RV1 cells (Additional file 6: Fig. S6D, E). A malignant signature comprising the 7 genes was hence developed for the quantification of the stemness-associated 5hmC gain of individual tumors. As expected, while the signature was positively associated with the previously established hyper-hydroxymethylation signature (Fig. 5B), it was also indicative of the PFS and the Gleason score in the TCGA PRAD cohort (Fig. 5C, D and Additional file 6: Fig. S6F). Given that tumors are heterogeneous entities, we next explored the presence of tumor cells with stemness-associated 5hmC gain in prostate cancer. The Scissor [33] algorithm was applied to a published single-cell RNA-seq dataset of prostate cancer comprising basal/intermediate, luminal and CellCycle subtypes of tumor cells (Fig. 5E) [34]. A total of 1248 Scissor-positive (Scissor^+^) tumor cells were subsequently identified, while the rest 1059 tumor cells were assigned as Scissor-negative (Scissor^−^) (Fig. 5F). The Scissor^+^ rate was higher in the CellCycle subtype than the basal/intermediate subtype (Fig. 5G), which is consistent with previous reports that CellCycle and basal/intermediate cells were associated with worse and better clinical outcomes, respectively [34]. Additionally, Scissor^+^ tumor cells showed both a higher malignant score and a higher adult stem cell signature than Scissor^−^ cells (Fig. 5H). Gene set enrichment analysis also suggested that stem cell-related pathways, such as hypoxia, MTORC1 signaling, Notch signaling and somatic stem cell maintenance, were enriched in Scissor^+^ tumor cells (Fig. 5I). Collectively, the identification of Scissor^+^ tumor cells emphasized the clinical relevance of the malignant score and confirmed the presence of tumor cell subpopulations with stemness-associated 5hmC gain in prostate tumors.

### Inhibition of the growth of tumor stem cell-like cells by vitamin C

**Fig. 6:**
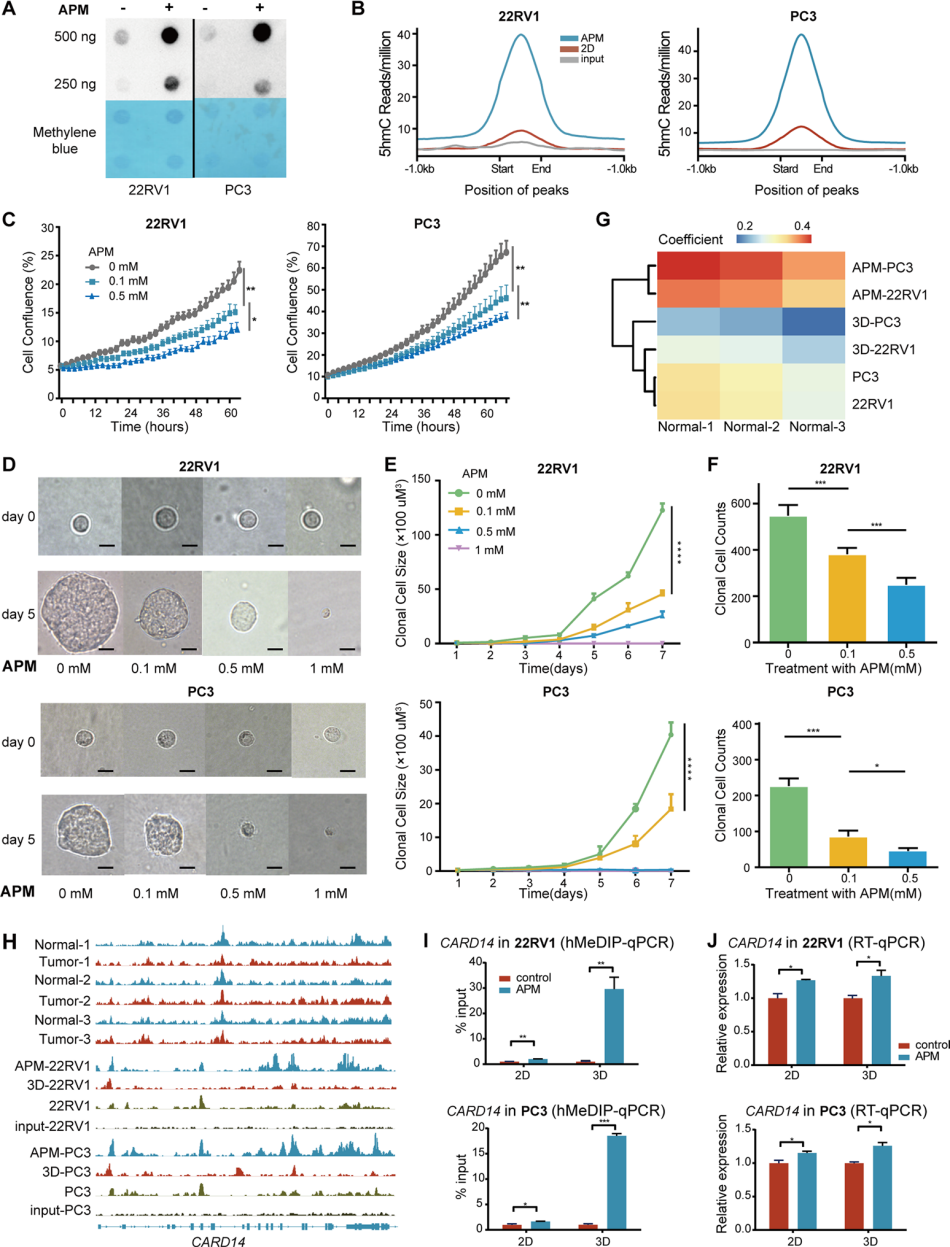
Inhibition of the growth of tumor stem cell-like cells by vitamin C. **A** Dot blot of 5hmC levels in prostate carcinoma cell lines 22RV1 and PC3 receiving APM treatment and vehicle-treated control cells. **B** 5hmC density at hyper-DhMRs shared by APM-treated 22RV1 (left) and PC3 (right) cells. **C** Proliferation curves of 22RV1 and PC3 cells in medium supplemented with increasing concentrations of APM. **D**, **E** 22RV1 and PC3 spheroids cultivated in 3D soft gel with the presence of increasing concentrations of APM at day 0 and day 5 (**E**) and the growth kinetics over 7 consecutive days (**F**); scale bar = 10 µm. **F** Bar plots showing the influence of APM treatment for 7 days on the number of 22RV1 and PC3 spheroids. **G** Heatmap of Pearson correlation values that were generated on the 5hmC patterns at 100-bp genomic windows of the indicated samples. **H** Genome browser tracks depicting 5hmC levels of *CARD14* in normal prostate samples, prostate cancer cells, prostate cancer cells treated with APM and prostate cancer cells cultured at 3D systems. **I, J** Bar plots displaying 5hmC (**I**) and gene expression levels (**J**) of *CARD14* in APM- and vehicle-treated 22RV1 and PC3 cells cultured in 2D and 3D systems. In (**C**), (**F**), (**G**), (**I**) and (**J)**, error bars represent mean ± standard deviation. *P* values were produced with *t* test. ****P < 0.0001; ***P < 0.001; **P < 0.01;*P < 0.05

We recently showed that the oxidation-resistant vitamin C derivative, APM, can restore the 5hmC pattern by acting as a cofactor of TETs in kidney cancer and bladder cancer [3]. However, it remains unclear whether vitamin C can also inhibit prostate cancer cell growth especially cancer stem cell-like cells by reprogramming 5hmC. As anticipated, APM treatment restored the 5hmC level and inhibited the proliferation of PC3 and 22RV1 cells (Fig. 6A–C, Additional file 7: Fig. S7A, B). Interestingly, in the 3D culture system, treatment of prostate cancer cells with APM impaired not only the initiation of 3D tumor spheres but also the growth of established ones in a dose-dependent manner (Fig. 6D–F), suggesting that APM may be able to inhibit the proliferation of prostate cancer stem-like cells.

To investigate whether any epigenetic events were involved in this process, we performed hMeDIP-seq, which in turn revealed that APM-treated PC3 and 22RV1 cells acquired 5hmC landscapes more similar to normal prostate tissues (Fig. 6G). For example, the 5hmC level and expression of *CARD14* were remarkably increased upon APM treatment to match what was observed in the normal prostate (Fig. 6H–J). Of note, genes restoring 5hmC level upon APM treatment were enriched in apoptosis-related cellular processes (Fig. 6H and Additional file 7: Fig. S7C), implying a pivotal role of apoptosis in APM-induced inhibition. The result of Gene Ontology (GO) analysis on RNA-seq data of APM-treated cells and 3D gel cultured cells also showed activated apoptotic process in APM-treated 22RV1 and PC3 cell lines, but negative regulation in 3D-cultured cell lines (Additional file 7: Fig. S7D), suggesting the potential regulatory role of APM in apoptosis-related processes. Furthermore, we performed flow cytometry analysis, which confirmed that APM treatment increased the proportion of apoptotic prostate cancer cells in both 2D and 3D culture systems (Additional file 7: Fig. S7E, F). Collectively, these results suggested that APM could be novel epigenetic therapeutics targeting cancer stem cell-like cells and inducing apoptosis for prostate cancers.

## Discussion

Advancing our understanding of the molecular underpinnings of aggressive malignancies is critical for discovering new therapeutic strategies, prognostic signatures and biomarkers. In this study, taking advantage of a highly sensitive 5hmC enrichment technology, hMeDIP-seq, we identified that both gain of 5hmC and loss of 5hmC occurred during GU tumorigenesis. Functional annotation analysis further showed that genes with 5hmC gain were enriched in stemness-, hypoxia- and immune-related pathways, while genes with 5hmC loss were enriched in proliferation, metabolism and cell adhesion pathways. The 5hmC alteration patterns were consistent with the scenario that tumorigenesis is a process of gain of properties of stem/progenitor cells and gradual loss of original cell identity (Graphical Abstract).

Previous studies, including ours, have comprehensively shown that loss of 5hmC is an epigenetic hallmark of cancers [2, 3]. However, to the best of our knowledge, regional gain of 5hmC was only clearly stated in a study of pancreatic cancer [48] and some genes with gain of 5hmC in certain cancers excluding GU cancers [49–51]. Moreover, several studies have shown that 5hmC modifications in circulating cell-free DNA are diagnostic markers for human cancers [4, 49, 52]. We noticed that all of these studies used a highly sensitive and enrichment technology to generate 5hmC patterns in tumor tissues or cell-free DNA from tumor patients. Thus, we hypothesized that cancer-associated 5hmC gain obtained from bulk tissues might be contributed by a small proportion of highly aggressive cancer stem cells within the tumor tissues, which might be underdetectable in studies using genome-wide single-nucleotide resolution TAB-seq technology [2].

Notably, we demonstrated 5hmC gain in 3D fibrin gel-induced cancer stem cell-like cells. Moreover, we confirmed that the gene sets with the co-occurrence of 5hmC gain and transcriptional up-regulation were associated with worse prognosis in prostate cancers. However, we observed that the expression change of TET enzymes in tumor tissue and cell lines is different, and *TET2* is down-regulated in cell lines of GU cancers but not in the tumor tissues (Additional file 8: Fig. S8A, B). Previously, we have shown APM as a cofactor of TETs to restore 5hmC in renal and bladder cancer cell lines [3, 53]. Nonetheless, it remains unclear whether vitamin C can inhibit prostate cancer growth especially cancer stem cell-like cells by reprogramming 5hmC. In this study, we found that APM treatment could inhibit the proliferation of prostate cancer cells and restore the 5hmC level similar to normal prostate tissues. Importantly, it can also suppress the proliferation of cancer stem-like cells and induces apoptosis in prostate cancer cell lines. Collectively, we confirmed that both 5hmC gain and 5hmC loss were epigenetic hallmarks of cancers, 5hmC gain might be contributed by highly aggressive cancer stem cells, and vitamin C might serve as an epigenetic differentiation therapy by targeting cancer stem cells. Of course, cell-type-specific 5hmC profiling of tumor-associated tissues and using appropriate cell-type markers would reveal the extent and distribution of the cell specificity of these modifications and shed further light on the properties of the source cells that contribute to the cancer-associated 5hmC changes observed in bulk tissue. We intend to pursue these future research directions.

## Conclusions

Overall, we showed that genes with 5hmC gain in GU tumors were associated with stemness, hypoxia or cross talk with the immune system, indicating that 5hmC alteration could serve as a hallmark signature for the tumorigenesis of GU cancers. Meanwhile, 5hmC gain in soft fibrin gel-induced 3D tumor spheres was then captured, and the associated genes showed enrichment in biological pathways related to stem cell maintenance and tumor progression. Finally, we observed restoration of 5hmC level in apoptosis pathways and inhibited proliferation in prostate cancer cell lines by treating with APM, a vitamin C derivative and a well-known reprogramming drug.

## Supplementary Information

**Additional** **file1:** **Fig. S1**. Genome-wide profiling of 5hmC in genitourinary tissues (related to Fig. 1).

**Additional** **file2:** **Fig. S2**. The distribution of the DhMR in the genitourinary normal tissues (related to Fig. 1).

**Additional** **file3:** **Fig. S3**. Summary of the DhMR location distribution in the genitourinary tumor genome (related to Fig. 2).

**Additional** **file4:** **Fig. S4**. The expression of DhMRs affected genes in the paired normal and tumor tissues of KIRC, PRAD and UC cohorts from the TCGA.

**Additional** **file5:** **Fig. S5**. Genes associated with hypo-5hmC alterations in genitourinary cancers clinical outcome (related to Fig. 3).

**Additional** **file6:** **Fig. S6**. The 3D-cultured cell signatures associated with aggressive epithelial cancer phenotypes (related to Fig. 5).

**Additional** **file7:** **Fig. S7**. APM-induced 5hmC gain in prostate cancer cells (related to Fig. 6).

**Additional** **file8:** **Fig. S8**. The expression of TETs in GU cancer tissues and GU cells.

**Additional** **file9:** **Table S1. **The primers of hMeDIP-qPCR and RT-qPCR.

**Additional** **file10:** **Table S2. **DhMRs position of each genitourinary tissues.

**Additional** **file11: Table S3. **The signatures of tissue hypo or hyper signatures.

**Additional** **file12:** **Table S4. **Analysis of hMeDIP-seq and RNA-seq data of prostate cancer cells.

**Additional** **file13:**  **Table S5. **Summary of hMeDIP-seq and RNA-seq data.

## Data Availability

The raw sequence data reported in this paper have been deposited in the Genome Sequence Archive [54] of the National Genomics Data Center [55], Beijing Institute of Genomics (China National Center for Bioinformation), Chinese Academy of Sciences, under accession number CRA003478 and are publicly accessible at https://ngdc.cncb.ac.cn/gsa. A summary of RNA-seq and hMeDIP-seq data is presented in Additional file 13: Table S5.
