# Supplementary material for: Regional gain and global loss of 5-hydroxymethylcytosine coexist in genitourinary cancers and regulate different oncogenic pathways
Source: Clin Epigenetics. 2022 Sep 20;14:117. doi: 10.1186/s13148-022-01333-4 (PMC9491006; doi:10.1186/s13148-022-01333-4)
Supplement: Supplementary file 1 — Additional file1: Fig. S1. Genome-wide profiling of 5hmC in genitourinary tissues (related to Fig. 1). [file 13148_2022_1333_MOESM1_ESM.docx]

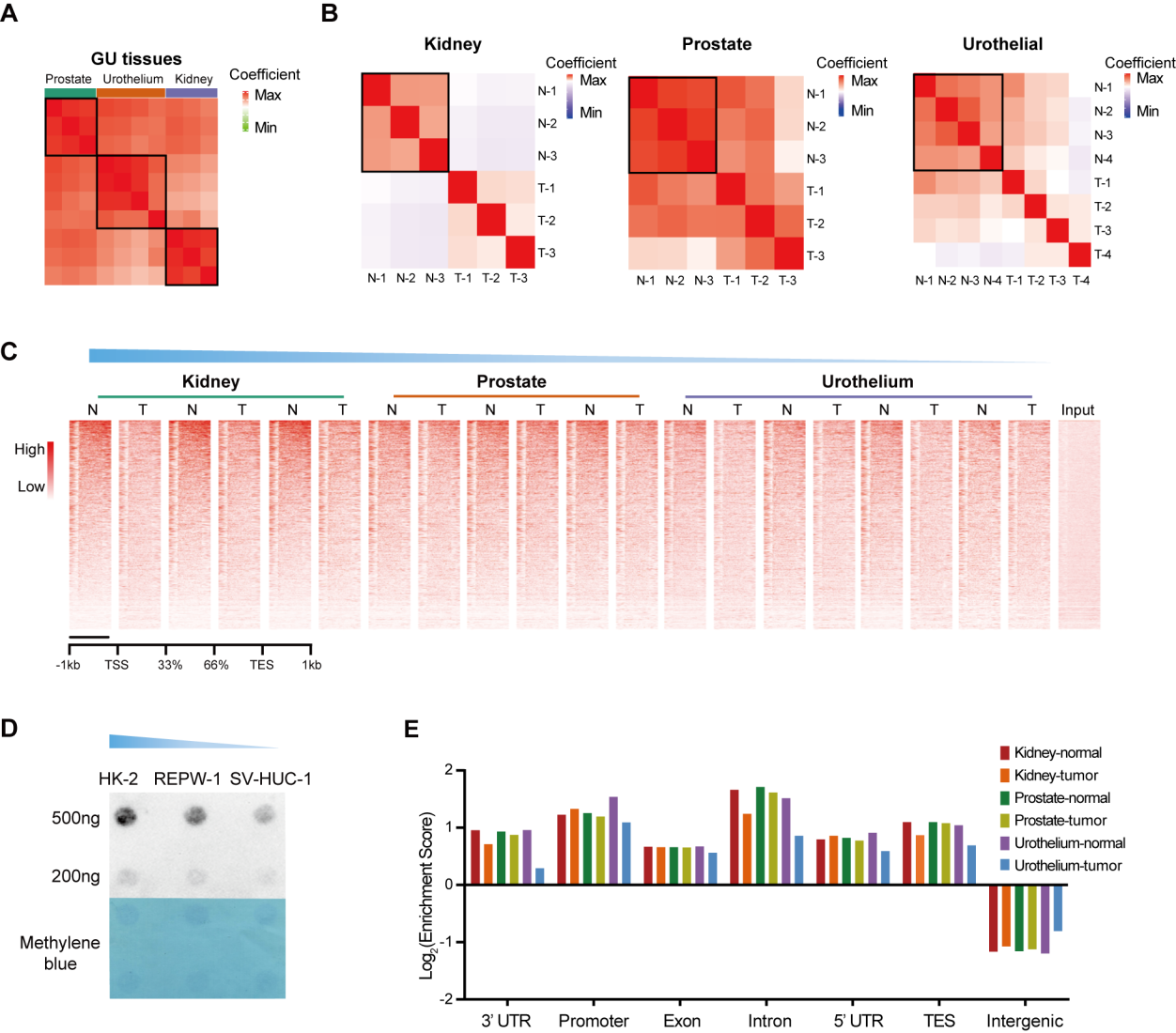


**Additional Fig 1.** **Genome-wide profiling of 5hmC in genitourinary tissues (related to Figure 1)**

**A-B.** Heatmaps showing the clustering of normal genitourinary samples based on normalized read counts of each sample with hMeDIP-seq data (A) and the clustering of genitourinary normal and tumors based on normalized read counts of each sample with hMeDIP-seq data (B). Colors indicate normalized read counts of hMeDIP-seq.

**C.** Meta-gene plot of 5hmC profiles in genitourinary tissues. The color range indicates log_2_ (TPM+1) values. TSS, transcription start site. TES, transcription end site.

**D.** Dot blot showing distinct 5hmC levels in normal cell lines (top). Methylene blue (MB) staining was used as a DNA loading control (bottom).

**E.** Bar plot showing the enrichment score of 5hmC peaks at 3' and 5' untranslated regions (3' UTR and 5' UTR), promoters, exons, introns, transcription end sites (TES), and intergenic regions in the genitourinary tissues.
