# Supplementary material for: Regional gain and global loss of 5-hydroxymethylcytosine coexist in genitourinary cancers and regulate different oncogenic pathways
Source: Clin Epigenetics. 2022 Sep 20;14:117. doi: 10.1186/s13148-022-01333-4 (PMC9491006; doi:10.1186/s13148-022-01333-4)
Supplement: Supplementary file 2 — Additional file2: Fig. S2. The distribution of the DhMR in the genitourinary normal tissues (related to Fig. 1). [file 13148_2022_1333_MOESM2_ESM.docx]

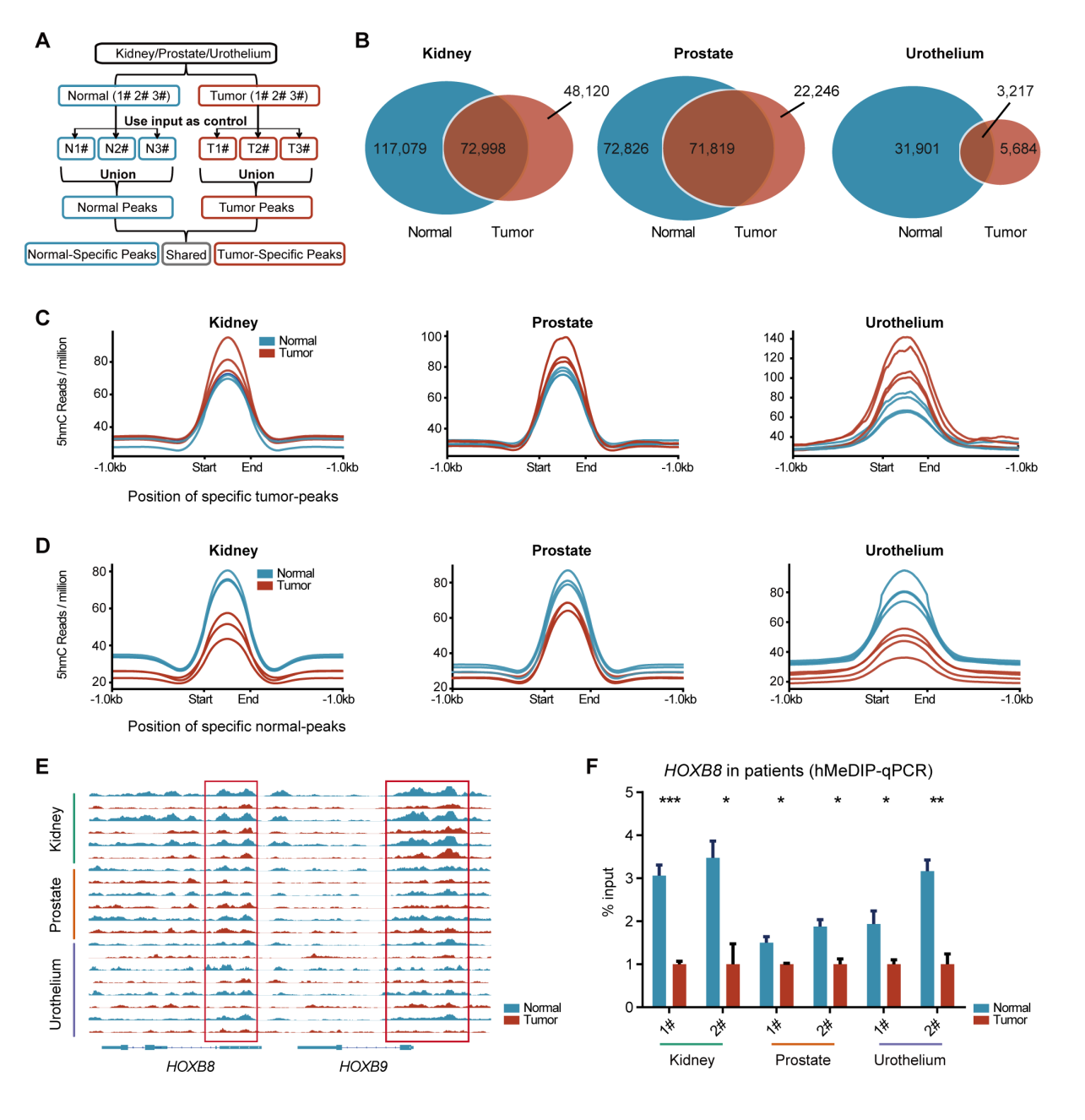


**Additional Fig 2. The distribution of the specific 5hmC peaks in the genitourinary normal and tumor tissues (related to Figure 1)**

1. The flow chart of the tumor-specific or normal-specific DhMRs from the genitourinary tissues.

**B.** Numbers of specific and shared 5hmC peaks between normal and tumor tissues of kidney (left), prostate (middle) and urothelium (right).

**C.** Average normalized 5hmC read counts across tumor kidney (left), prostate (middle) and urothelium (right) tissue-enriched peaks. Blue indicates normal tissues; red indicates tumor tissues.

**D.** Genome browser tracks depicting 5hmC levels of normal kidney (left), prostate (middle) and urothelium (right) tissue-enriched peaks.

**E.** Genome browser tracks depicting the 5hmC level of *HOXB8* and *HOXB9* in healthy and cancerous genitourinary tissues including the kidney, the prostate and the urothelium.

**F.** Bar plot showing the 5hmC level of *HOXB8* measured by hMeDIP-qPCR in matched normal and tumor samples of the kidney, the prostate and the urothelium. Primers were designed at the positions indicated by red boxes in (G). Error bars represent mean ± standard deviation. *P* values were produced with *t*-test.
