# Supplementary material for: Regional gain and global loss of 5-hydroxymethylcytosine coexist in genitourinary cancers and regulate different oncogenic pathways
Source: Clin Epigenetics. 2022 Sep 20;14:117. doi: 10.1186/s13148-022-01333-4 (PMC9491006; doi:10.1186/s13148-022-01333-4)
Supplement: Supplementary file 3 — Additional file3: Fig. S3. Summary of the DhMR location distribution in the genitourinary tumor genome (related to Fig. 2). [file 13148_2022_1333_MOESM3_ESM.docx]

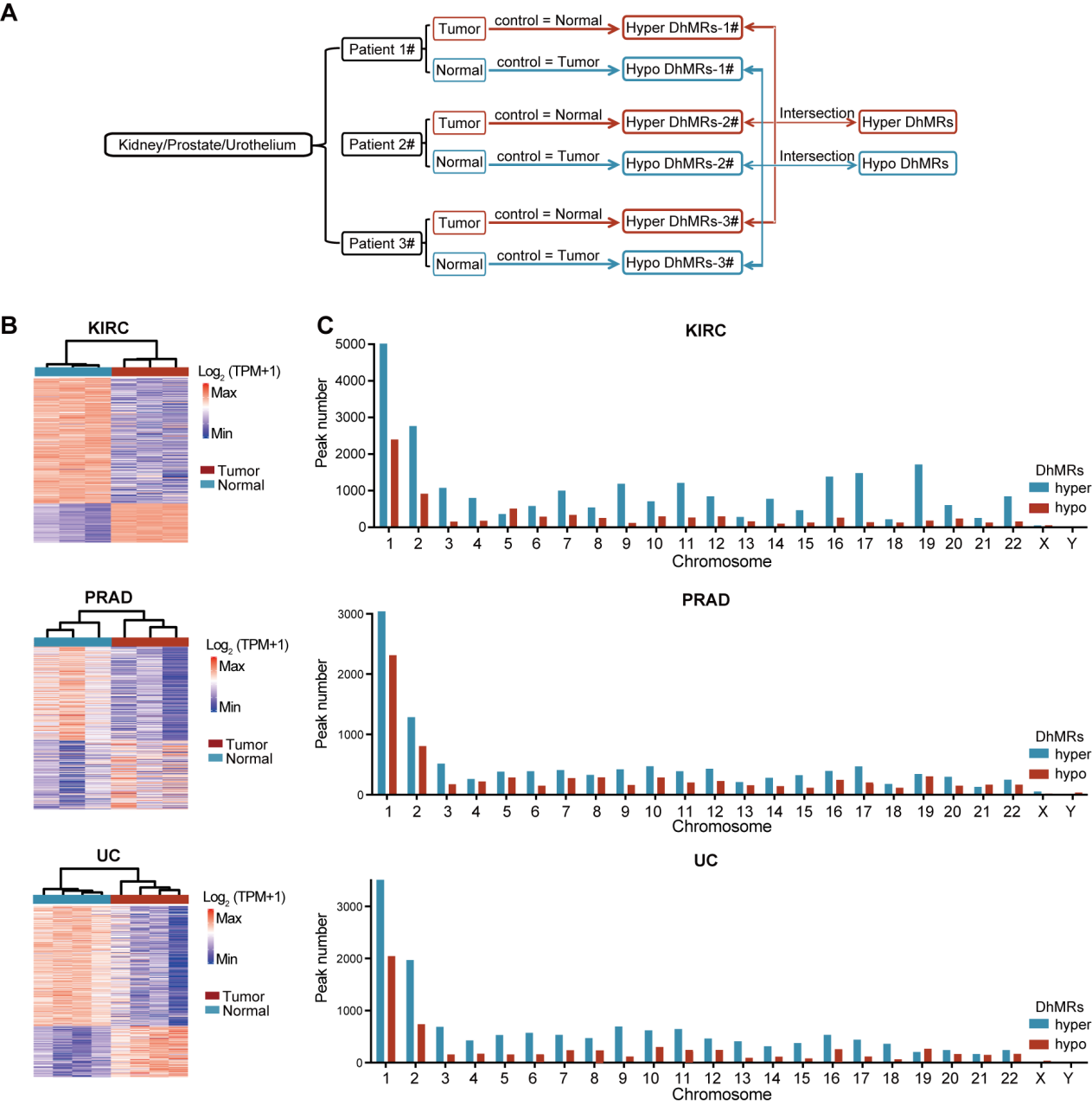


**Additional Fig 3. Summary of the DhMR location distribution in the genitourinary tumor genome and expression level of the affected genes (related to Figure 2)**

**A.** The flow chart of the established (hyper-) and absent (hypo-) DhMRs from the genitourinary system derived from each patients.

**B.** Heatmaps showing the clustering of normal and tumor kidney (up), prostate (middle) and urothelium (down) tissues based on hypo-DhMRs. Colors indicate normalized read counts of hMeDIP-seq.

**C.** Bar plot of numbers of GU cancers-specific DhMRs on each chromosome.
