# Supplementary material for: Regional gain and global loss of 5-hydroxymethylcytosine coexist in genitourinary cancers and regulate different oncogenic pathways
Source: Clin Epigenetics. 2022 Sep 20;14:117. doi: 10.1186/s13148-022-01333-4 (PMC9491006; doi:10.1186/s13148-022-01333-4)
Supplement: Supplementary file 4 — Additional file4: Fig. S4. The expression of DhMRs affected genes in the paired normal and tumor tissues of KIRC, PRAD and UC cohorts from the TCGA. [file 13148_2022_1333_MOESM4_ESM.docx]

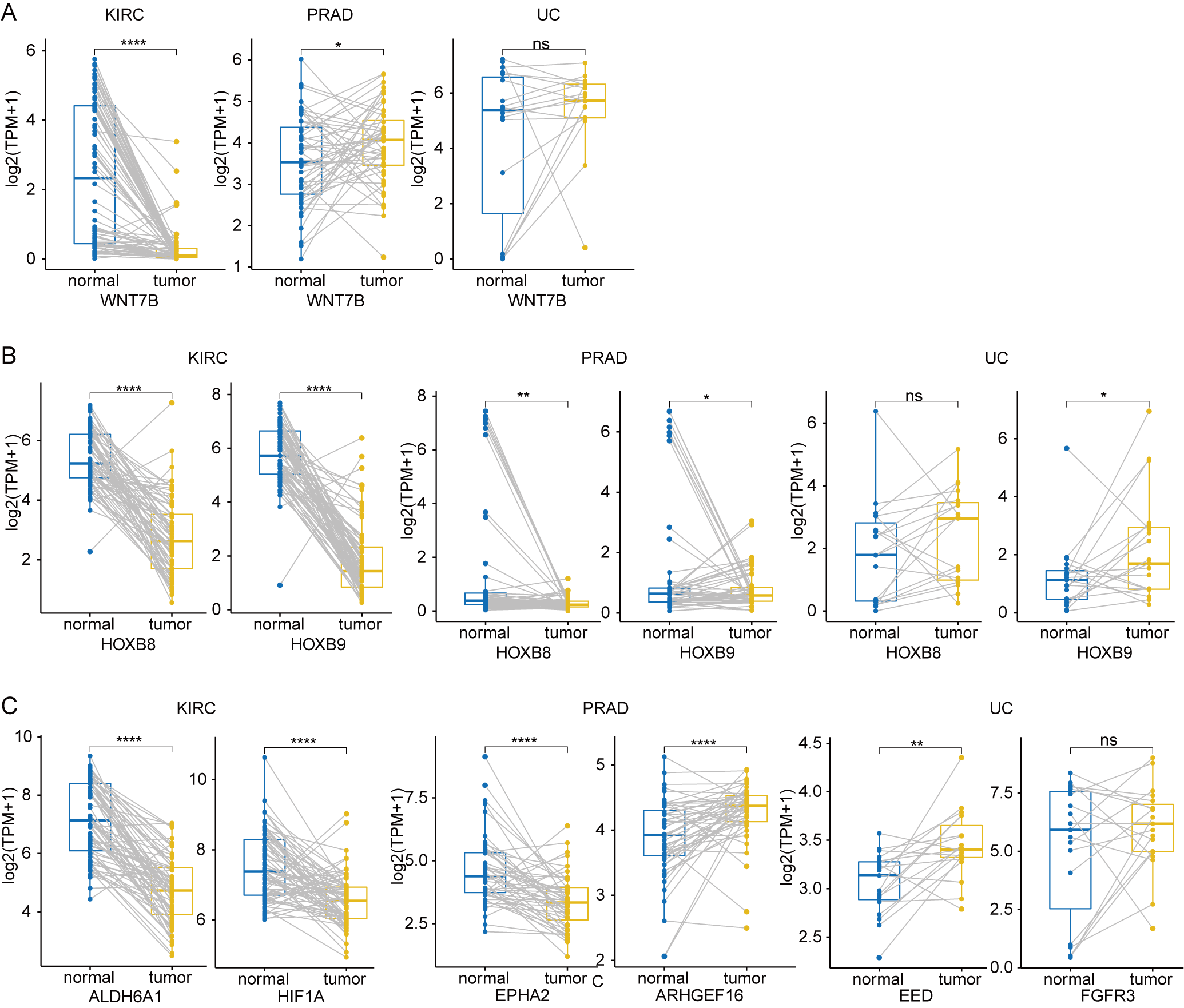


**Additional Fig 4.** **The expression of DhMRs affected genes in the paired normal and tumor tissues of KIRC, PRAD and UC cohorts from the TCGA.**

**A-B.** Box plot displaying the expression of WNT7B (A) and HOXB8, HOXB9 (B) in tumor tissues compared to in paired normal tissues in the TCGA KIRC, PRAD and BLCA cohorts.

**C.** Box plot showing the expression of ALDH6A1, EPHA2, EED, HIF1A, ARHGEF16 and FGFR3 in tumor tissues compared to in paired normal tissues in the TCGA KIRC, PRAD and BLCA cohorts.

In (A-C) error bars represent mean ± standard deviation. P values were produced with t-test. ***P < 0.001; **P < 0.01;*P < 0.05.
