# Supplementary material for: Regional gain and global loss of 5-hydroxymethylcytosine coexist in genitourinary cancers and regulate different oncogenic pathways
Source: Clin Epigenetics. 2022 Sep 20;14:117. doi: 10.1186/s13148-022-01333-4 (PMC9491006; doi:10.1186/s13148-022-01333-4)
Supplement: Supplementary file 5 — Additional file5: Fig. S5. Genes associated with hypo-5hmC alterations in genitourinary cancers clinical outcome (related to Fig. 3). [file 13148_2022_1333_MOESM5_ESM.docx]

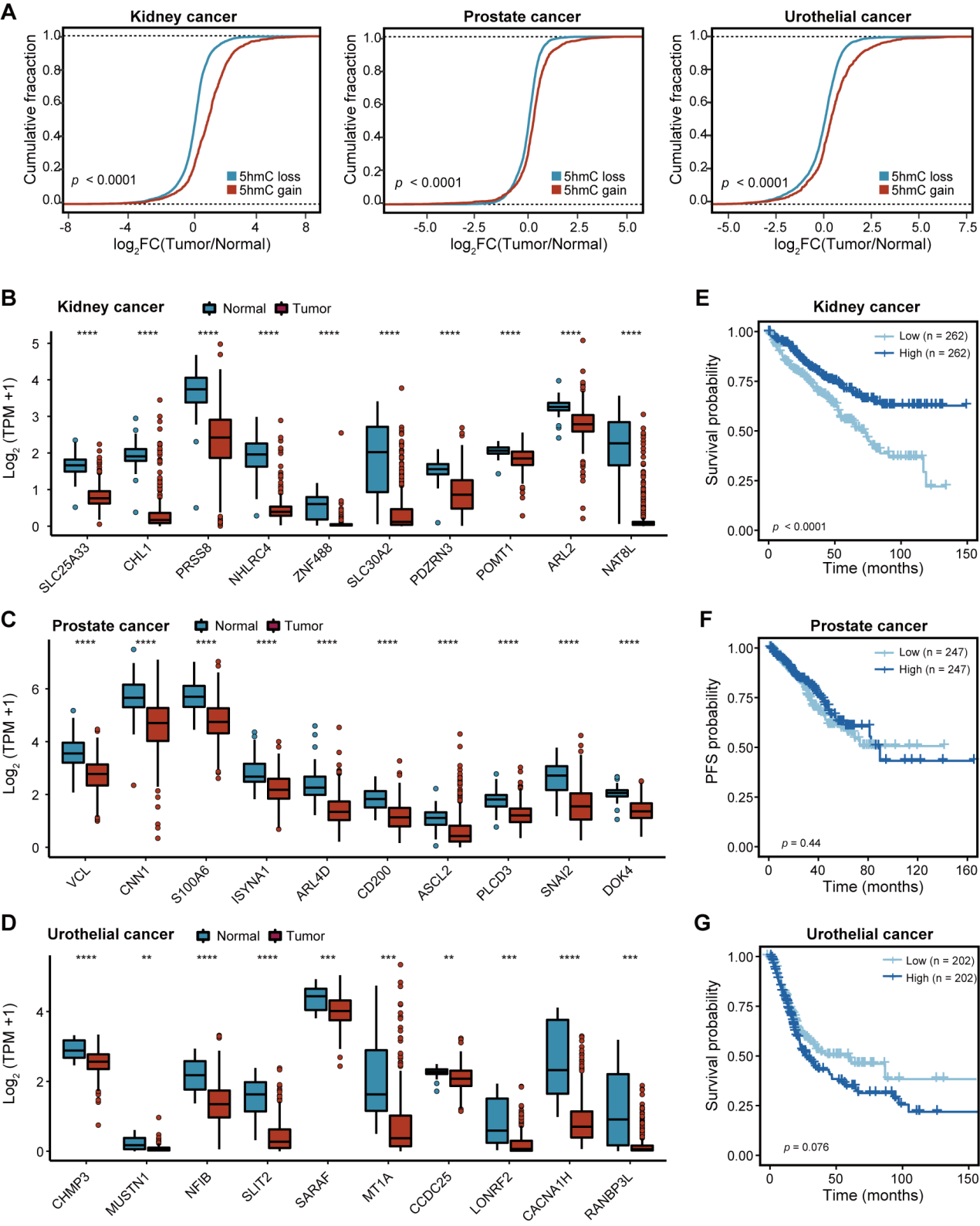


**Additional Fig 5. Genes associated with hypo-5hmC alterations in genitourinary cancers clinical outcome (related to Figure 3)**

**A.** Cumulative curve of transcription levels in kidney (left), prostate (middle) and urothelium (right) tissue DhMRs.

**B-D.** Box plot displaying the expression of hypo-DhMR associated genes with the most transcriptional changes compared to normal tissues in the TCGA KIRC (B), PRAD (C) and BLCA (D) cohorts. *P* values were determined by the Student’s *t*-test. *****P* < 0.0001; ****P* < 0.001; ***P* < 0.01;**P* < 0.05.

**E-G.** Kaplan-Meier plot of the relationship between patient overall survival, progression-free survival and hyper-hydroxymethylated signature scores within the TCGA KIRC (E), PRAD (F) and BLCA (G) cohorts. Patients were stratified by the score median. P values were produced using log rank test.
