# Supplementary material for: Regional gain and global loss of 5-hydroxymethylcytosine coexist in genitourinary cancers and regulate different oncogenic pathways
Source: Clin Epigenetics. 2022 Sep 20;14:117. doi: 10.1186/s13148-022-01333-4 (PMC9491006; doi:10.1186/s13148-022-01333-4)
Supplement: Supplementary file 6 — Additional file6: Fig. S6. The 3D-cultured cell signatures associated with aggressive epithelial cancer phenotypes (related to Fig. 5). [file 13148_2022_1333_MOESM6_ESM.docx]

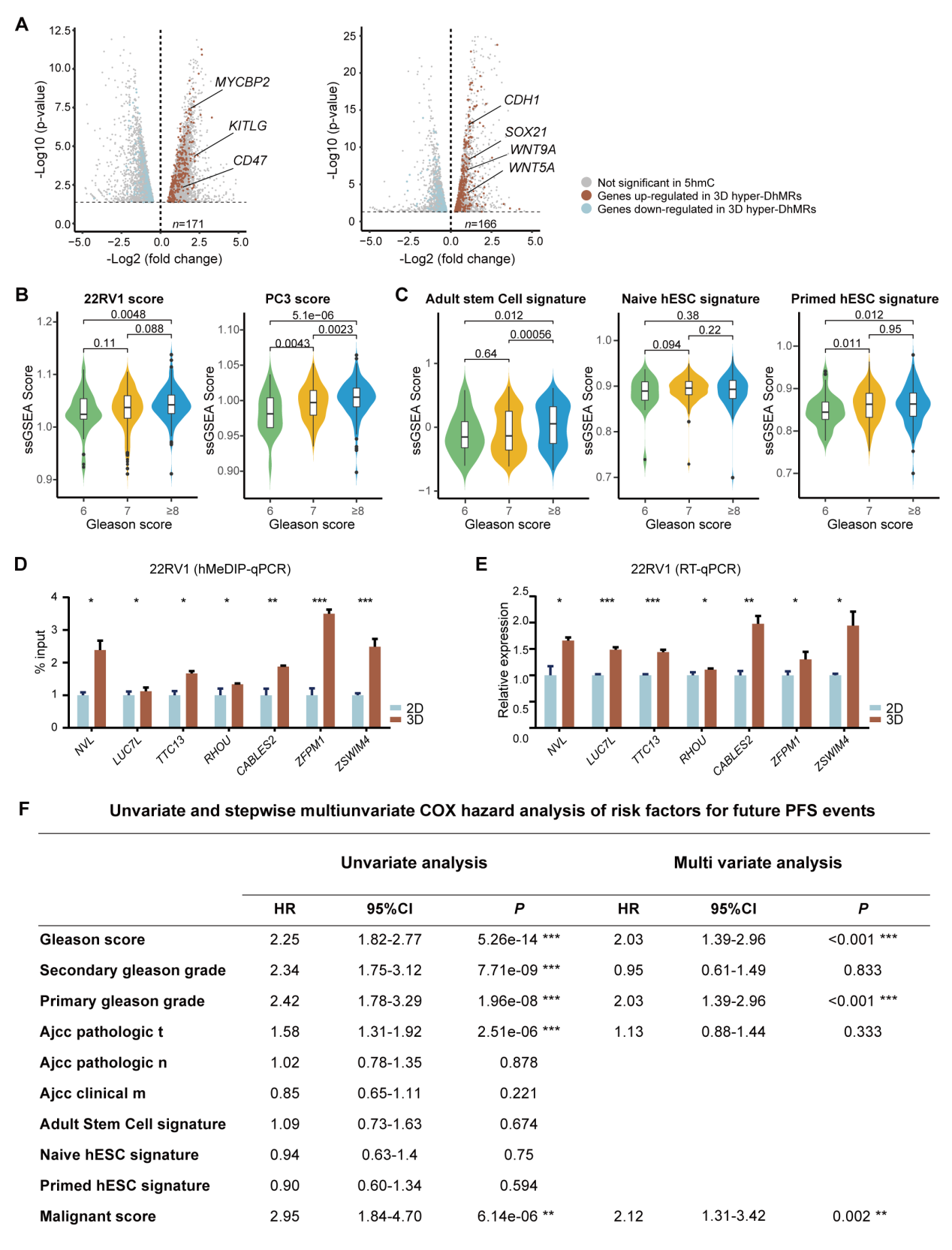


**Additional Fig 6. The 3D-cultured cell signatures associated with aggressive epithelial cancer phenotypes (related to Figure 5)**

**A**. Differentially expressed genes in 3D-cultured 22RV1 (left) and PC3 (right) cells. Blue dots: 3D-enriched DhMRs with increasing gene expression. Red dots: 3D-enriched DhMRs with decreasing gene expression.

**B**. Violin plots showing estimated 3D-cultured cell signatures of the PC3 signature (left) and 22RV1 (right) signature scores of patients stratified by the Gleason score.

**C.** Violin plots showing estimated the aggressive signature, including the adult stem cell signature (left), naive hESC signature (middle) and primed hESC signature (right) scores of patients stratified by the Gleason score.

**D-E.** Quantification of the 7 malignant genes expression in 22RV1 and PC3 cell lines by real-time PCR (D) and hMeDIP-seq (E). Cells cultured in 2D rigid dishes were used as control.

**F.** Unvariate and stepwise multiunvariate COX hazard analysis of risk factors for future PFS events.

In (B-F) error bars represent mean ± standard deviation. *P* values were produced with *t*-test. ****P* < 0.001; ***P* < 0.01;**P* < 0.05.
