# Supplementary material for: Regional gain and global loss of 5-hydroxymethylcytosine coexist in genitourinary cancers and regulate different oncogenic pathways
Source: Clin Epigenetics. 2022 Sep 20;14:117. doi: 10.1186/s13148-022-01333-4 (PMC9491006; doi:10.1186/s13148-022-01333-4)
Supplement: Supplementary file 7 — Additional file7: Fig. S7. APM-induced 5hmC gain in prostate cancer cells (related to Fig. 6). [file 13148_2022_1333_MOESM7_ESM.docx]

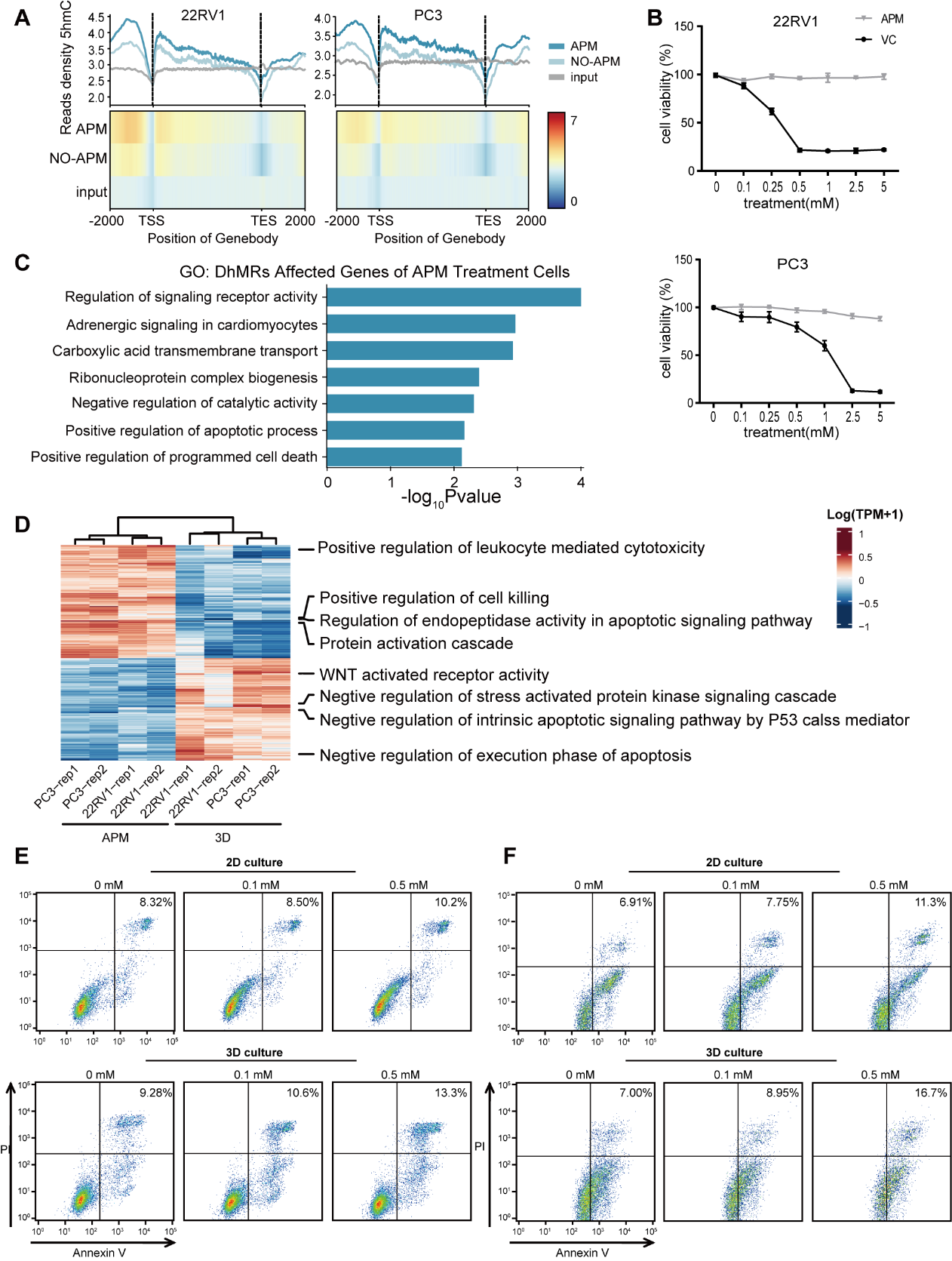


**Additional Fig 7. APM-induced apoptosis in prostate cancer cells (related to Figure 6)**

1. HMeDIP-seq (H) showing the 5hmC alteration introduced by the 3D-culture in prostate carcinoma cell lines 22RV1 and PC3.
2. The cytotoxic effect of vitamin C and APM on prostate cancer cells of 22RV1 (up) and PC3 cells (down).
3. Gene Ontology (GO) analysis of the specific DhMRs affecting genes of APM treated cells.
4. Gene Ontology (GO) analysis of the transcriptomes data of APM treated cells and 3D gel cultured cells.

**E-F.** Apoptosis assay of PC3 cells (E) and 22RV1 (F) at varying concentrations with APM.
