# Supplementary material for: Regional gain and global loss of 5-hydroxymethylcytosine coexist in genitourinary cancers and regulate different oncogenic pathways
Source: Clin Epigenetics. 2022 Sep 20;14:117. doi: 10.1186/s13148-022-01333-4 (PMC9491006; doi:10.1186/s13148-022-01333-4)
Supplement: Supplementary file 8 — Additional file8: Fig. S8. The expression of TETs in GU cancer tissues and GU cells. [file 13148_2022_1333_MOESM8_ESM.docx]

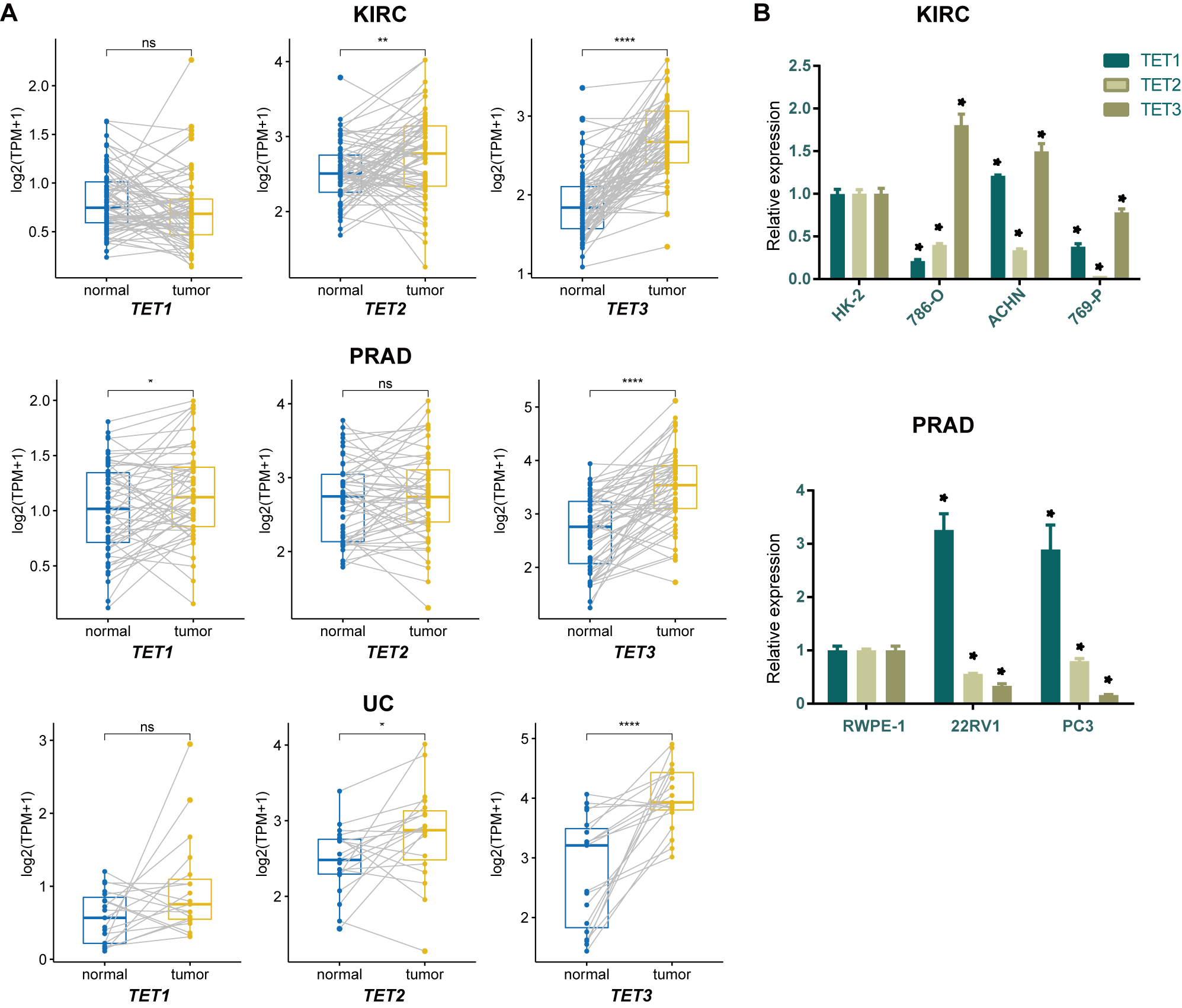


**Additional Fig 8.** **The expression of TETs in GU cancer tissues and GU cells.**

1. Box plot displaying the expression of TET1, TET2, and TET3 in tumor tissues compared to in the paired normal tissues in the TCGA KIRC, PRAD and BLCA cohorts.

**B.** Quantification of TET1, TET2, and TET3 in in the normal and cancer cell lines of kidney (up) and prostate (down) by real-time PCR. Normal cell lines were used as control.

In (A-B) error bars represent mean ± standard deviation. P values were produced with t-test. ***P < 0.001; **P < 0.01;*P < 0.05.
